# Antigenic Characterization of the HCMV gH/gL/gO and Pentamer Cell Entry Complexes Reveals Binding Sites for Potently Neutralizing Human Antibodies

**DOI:** 10.1371/journal.ppat.1005230

**Published:** 2015-10-20

**Authors:** Claudio Ciferri, Sumana Chandramouli, Alexander Leitner, Danilo Donnarumma, Michael A. Cianfrocco, Rachel Gerrein, Kristian Friedrich, Yukti Aggarwal, Giuseppe Palladino, Ruedi Aebersold, Nathalie Norais, Ethan C. Settembre, Andrea Carfi

**Affiliations:** 1 Novartis Vaccines (a GSK company), Cambridge, Massachusetts, United States of America; 2 ETH Zürich, Department of Biology, Institute of Molecular Systems Biology, Zürich, Switzerland; 3 Novartis Vaccines (a GSK company), Siena, Italy; 4 Harvard University, Department of Molecular and Cellular Biology, Cambridge, Massachusetts, United States of America; 5 Harvard Medical School, Department of Cell Biology, Boston, Massachusetts, United States of America; 6 Faculty of Science, University of Zurich, Zurich, Switzerland; Tufts University, UNITED STATES

## Abstract

Human Cytomegalovirus (HCMV) is a major cause of morbidity and mortality in transplant patients and in fetuses following congenital infection. The glycoprotein complexes gH/gL/gO and gH/gL/UL128/UL130/UL131A (Pentamer) are required for HCMV entry in fibroblasts and endothelial/epithelial cells, respectively, and are targeted by potently neutralizing antibodies in the infected host. Using purified soluble forms of gH/gL/gO and Pentamer as well as a panel of naturally elicited human monoclonal antibodies, we determined the location of key neutralizing epitopes on the gH/gL/gO and Pentamer surfaces. Mass Spectrometry (MS) coupled to Chemical Crosslinking or to Hydrogen Deuterium Exchange was used to define residues that are either in proximity or part of neutralizing epitopes on the glycoprotein complexes. We also determined the molecular architecture of the gH/gL/gO- and Pentamer-antibody complexes by Electron Microscopy (EM) and 3D reconstructions. The EM analysis revealed that the Pentamer specific neutralizing antibodies bind to two opposite surfaces of the complex, suggesting that they may neutralize infection by different mechanisms. Together, our data identify the location of neutralizing antibodies binding sites on the gH/gL/gO and Pentamer complexes and provide a framework for the development of antibodies and vaccines against HCMV.

## Author Summary

Human Cytomegalovirus (HCMV) is a double stranded DNA, enveloped virus infecting >60% of the population worldwide. Typically asymptomatic in healthy adults, HCMV infection causes morbidity and mortality in immunocompromised patients and is the most common viral cause of birth defects in industrialized countries. Despite more than 30 years of research, however, no vaccine against HCMV is available. HCMV utilizes two distinct glycoprotein complexes, gH/gL/gO and gH/gL/UL128/UL130/UL131A (Pentamer), to enter fibroblast and endothelial/epithelial cells, respectively and both are neutralizing antibodies targets. We used orthogonal techniques to study the interaction between gH/gL/gO or Pentamer and a panel of naturally occurring human neutralizing antibodies. The results of this analysis identify three neutralizing epitopes in gH, which are conserved in both glycoproteins complexes, and a different subset of five neutralizing sites in the UL128/Ul130/Ul131A (ULs) portion of the Pentamer. Moreover, EM analysis defines two distinct surfaces targeted by neutralizing antibodies on the ULs suggesting different neutralization mechanisms. Our results reveal regions of the gH/gL/gO and Pentamer complexes important for eliciting strong neutralizing responses in humans and for function in viral entry. Together our data will guide the development of therapeutic monoclonal antibodies and vaccines against HCMV.

## Introduction

Human Cytomegalovirus (HCMV), a member of the *Betaherpesvirinae* sub-family of *Herpesviridae*, infects 40–60% of the human adult population [1]. Similar to other herpesviruses, after primary infection, HCMV becomes latent and persists for the host’s life span. Reactivation is generally asymptomatic in immune-competent individuals. However, primary infection or reactivation can cause severe disease or death in immuno-suppressed hosts such as solid organ and hematopoietic stem cell (HSC) transplant patients and individuals with HIV infection [2–5]. HCMV is also the most common cause of viral induced birth defects affecting 0.2% of the newborns in industrialized countries [6–8]. For this reason the development of an effective HCMV vaccine able to prevent congenital infection was identified as a top priority by the Institute of Medicine [9–13]. Nevertheless, despite more than 20 years of vaccine research, there is no vaccine available against HCMV.

HCMV can infect a broad spectrum of cell types including epithelial and endothelial cells, fibroblasts, dendritic cells, neurons, and leukocytes [14,15]. The virus uses several envelope glycoprotein complexes to enter cells. Like other herpesviruses, glycoprotein B (gB), the viral fusion protein, and a gH/gL containing complex are required for HCMV cell entry [16,17]. Specifically, HCMV entry into epithelial and endothelial cells requires a pentameric glycoprotein complex (Pentamer) comprised of the gH, gL, UL128, UL130, and UL131A subunits (from now on referred to as ULs) [18,19] and is dependent on low pH [19]. Instead, entry into fibroblasts requires the gH/gL/gO complex and viral membrane fusion is thought to occur at the plasma membrane [20–23].

Recent data indicate that all HCMV strains contain gH/gL/gO and Pentamer complexes on the viral envelope and little, if any, unbound gH/gL [24]. Mutations in the *UL131A-UL128* gene locus occur spontaneously within just a few passages of wild-type (WT) HCMV in fibroblasts and are sufficient to eliminate epithelial/endothelial cell tropism [25]. Conversely, deletion of gO from the HCMV genome compromises virion assembly and replication in fibroblasts [26]. Of note, cell surface Pentamer over-expression prevents HCMV entry into epithelial cells, but not into fibroblasts, presumably through host protein sequestration, indicating the presence of a cell-type specific Pentamer-receptor [27].

We have recently described the biochemical characterization of HCMV gH/gL, gH/gL/gO and Pentamer and defined the overall architecture of each complex on its own or bound to a Fab fragment from the neutralizing antibody MSL-109 [28]. Electron microscopy (EM) data showed that like HSV-2 gH/gL, HCMV gH/gL adopts a boot shaped structure. A similar structure was seen when HCMV gH/gL is complexed with gO or with the ULs. The EM analysis also revealed that gO, in gH/gL/gO, and the ULs, in Pentamer, bind to the same site at the N-terminal end of the gH/gL heterodimer, thus forming mutually exclusive cell entry complexes. Consistent with these observations mass spectrometry (MS) studies demonstrated that the same cysteine in gL, C144, forms disulfide bridges with UL128-C162 in Pentamer, gO-C351 in gH/gL/gO or with the same cysteine in homodimers of gH/gL heterodimers. Notably, mutation of gL-C144S was sufficient to prevent formation of covalent complexes between gH/gL and either gO or UL128 in gH/gL/gO and Pentamer, respectively. The same mutation resulted in formation of monomeric gH/gL heterodimers [28].

Highly potent monoclonal antibodies targeting conformational epitopes of the Pentamer were initially isolated from the memory B-cell repertoire of HCMV immune donors and later from rabbits and mice immunized with an experimental vaccine virus in which the expression of the Pentamer was restored or an adjuvanted Pentamer protein, respectively [29–31]. These antibodies were a thousand-fold more potent than antibodies against gB or the gH/gL complex and were extraordinarily effective in neutralizing HCMV infection of epithelial and endothelial cells. Recently, different groups have demonstrated that immunization with adjuvanted Pentamer protein or vectors expressing the Pentamer elicit a strong neutralizing response in small animals and rhesus macaques [31–34].

Despite the fact that these data indicate that Pentamer represents a key antigenic target for HCMV vaccine development, limited mapping data are available to describe the sites on the Pentamer that are recognized by neutralizing antibodies and responsible for eliciting its potent neutralizing response. In this study we describe the interaction between HCMV gH/gL/gO and Pentamer with naturally-elicited potently neutralizing human monoclonal antibodies [29] using a combination of EM and MS. Our data identify HCMV gH/gL/gO and Pentamer epitopes important for generating strong neutralizing responses providing a framework for the development of effective HCMV vaccines and antibody therapeutics.

## Results

### Identification and characterization of the gH neutralizing antibody binding sites

Two neutralizing monoclonal antibodies, 13H11 and 3G16, isolated from immortalized memory B-cells of HCMV-immune donors have been shown to bind to HCMV glycoprotein H (gH) and prevent infection [29,35]. We initially investigated binding of gH/gL to these two antibodies as well as to MSL-109, an antibody isolated from the spleen of an HCMV seropositive individual [36,37], whose gH binding site we previously characterized [36–38]. We also generated Fab fragments for the three antibodies for additional binding and structural studies.

**Fig 1:**
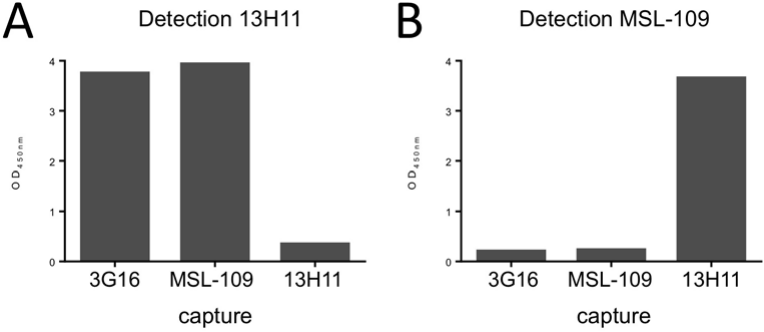
HCMV gH/gL antibody binding competition. HCMV gH/gL antibody binding competition in an ELISA assay. On the x-axis are the individual capturing monoclonal antibodies; on the y-axis is the optical density determined spectrophotometrically at 450 nm. Each graph represents the results from addition of one biotinylated detection antibody. Significant reduction in signal indicates competition between the capturing antibody and the detection antibody. **(A)** Binding of 3G16 or MSL-109 does not interfere with 13H11 binding indicating that 13H11 and 3G16 or MSL-109 do not compete for binding of gH. **(B)** Binding of 3G16 but not of 13H11, interferes with MSL-109 binding, indicating that 3G16 and MSL-109 compete.

An ELISA assay was initially used to study the interaction between the three monoclonal antibodies and the gH/gL homodimer (Fig 1). For each of the antibodies we were able to confirm gH/gL binding. In addition, we observed binding competition between MSL-109 and 3G16. 13H11, however, was able to form a ternary complex with either gH/gL/MSL-109 or gH/gL/3G16 (Fig 1). Equivalent results were obtained with the corresponding Fabs by gel shift assay using either gH/gL homodimer or the gH/gL-C144S mutant [28], which is mostly monomeric in solution, and the Fab fragments of the three antibodies (S1 Fig).

**Fig 2:**
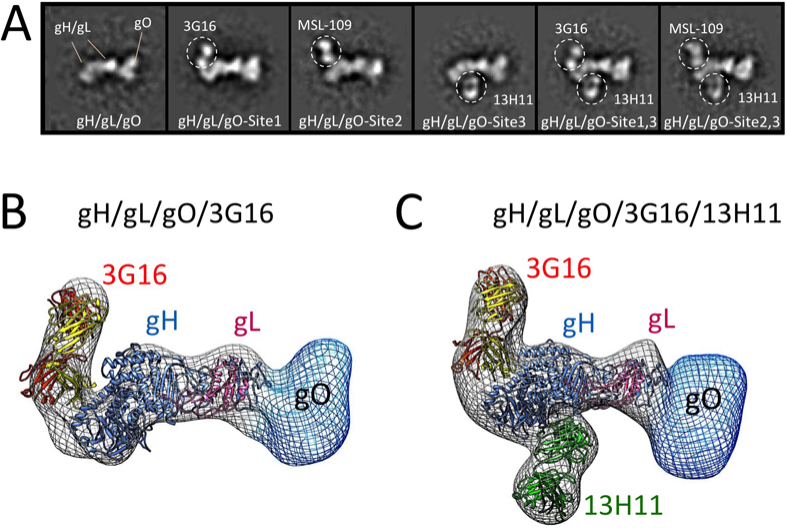
Epitope mapping of gH/gL-specific neutralizing antibodies by EM. **(A)** Reference-free 2D EM analysis reveals the binding sites of 3G16, MSL-109 and 13H11 neutralizing antibodies on the surface of gH/gL/gO. The analysis suggests that 3G16 and MSL-109 may sterically compete for binding gH/gL/gO while each of them can interact with gH/gL/gO at the same time as 13H11. **(B)** RCT reconstruction of the gH/gL/gO/3G16 complex. A model based on HSV-2 gH/gL crystal structure was fitted into the density map. Additional density emerging from the C-terminus of gH corresponds to 3G16. Density observed at the N-terminal region of gH/gL indicates gO’s position and shape. **(C)** RCT reconstruction of the gH/gL/gO/3G16/13H11 complex. Also in this case, a model of gH/gL was fitted into the density map. Additional densities emerging from the C-terminus and middle portion of gH corresponds to 3G16.and 13H11 respectively. Density at the N-terminal end of gH/gL accounts for gO.

To characterize the binding of these antibodies to gH, we reconstituted gH/gL/Fab and gH/gL/gO/Fab complexes and used EM and single particle analysis to identify the position of the specific antibody as compared to the unbound complex. Initial EM analysis revealed the same binding sites for the Fabs in both gH/gL-C144S monomer mutant and gH/gL/gO complexes. Only the latter was studied further due to the higher image quality (Fig 2A). EM analysis of the gH/gL/gO/3G16 complex demonstrated that the epitope of this antibody is localized in the C-terminal domain of gH. EM analysis of the previously characterized MSL-109 bound complex [28], showed that MSL-109 binds the external part of the gH/gL/gO complex ‘heel’ (Fig 2A). Comparison of the 3G16 and MSL-109 complexes suggest that the two Fabs cannot bind simultaneously to gH due to the spatial overlap of their constant regions. This result is consistent with the competition effect observed in ELISA and gel shift assay (Fig 1 and S1 Fig). Finally, analysis of 13H11-bound gH/gL/gO showed that the epitope of this antibody is localized internally at the gH/gL kinked region, opposite to the epitopes recognized by 3G16 and MSL-109. Analysis of gH/gL/gO bound to 3G16/13H11 or MSL-109/13H11 further confirms these conclusions (Fig 2A).

To support the results of our 2D EM analysis of the gH/gL/gO/Fab complexes, we determined a 3D-reconstruction of gH/gL/gO bound to 3G16 Fab and to both 3G16 and 13H11 Fabs (Fig 2B and 2C). Since these complexes adopted a strong preferential view we calculated the 3D structure using the Random Conical Tilt (RCT) method to ~19 Å and 29 Å resolution, respectively [39]. The reconstructed density maps of the gH/gL/gO-Fab complexes allowed fitting of an HCMV gH/gL model based on the HSV gH/gL crystal structure [40]. Models obtained for the 3G16 and 13H11 Fab fitted well into the density map and were located respectively proximal to the gH C-terminal domain and close to the gH-kinked region opposite to 3G16 and MSL-109. Additional density emerging from the N-terminal region of gH/gL describes the overall shape of the gO subunit as we previously reported [28].

### Localization of gH residues involved in antibody interaction by hydrogen-deuterium exchange coupled to MS

Hydrogen-deuterium exchange coupled to MS (HDX-MS) was used to identify residues that are part of the gH/gL epitopes targeted by 3G16 and 13H11 Fabs. To simplify the MS analysis, we expressed the monomeric gH/gL-C144S mutant in HEK293S GnTI^-/-^ cells as previously described [28]. The purified complex was deuterated either alone or in the presence of each Fab and the averaged deuterium exchange behaviors of 118 overlapping peptides of gH and gL were investigated. No gL peptides were observed having a difference in deuterium incorporation when gH/gL was bound to any of the Fabs confirming that these Fabs bind the gH subunit.

**Fig 3:**
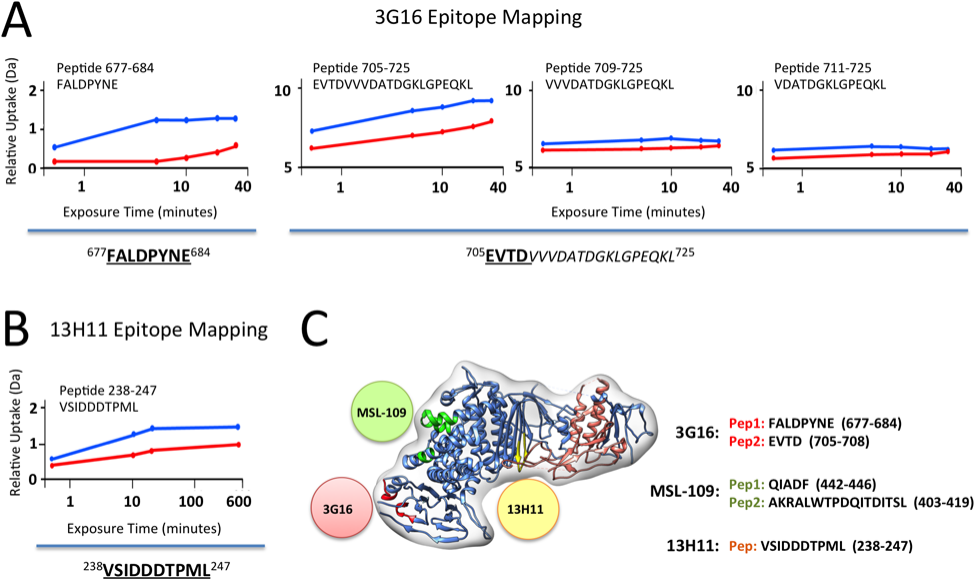
gH/gL epitope mapping by HDX-MS. Curves of peptide deuterium uptake in the presence and absence of Fab are reported in red and blue, respectively. **(A)** Analysis of 3G16 binding identifies two peptides in the C-terminal region of gH presenting a deuterium incorporation reduction upon binding (peptides 677–684 and 705–725). The portion of the epitope present in the peptide 705–725 is narrowed to the sequence 705–708 since the two peptides 709–725 and 711–725 do not present any difference in deuterium uptake upon binding. **(B)** Analysis of 13H11 binding identifies a single peptide localized to the central portion of gH (peptide 238–247). **(C)** HCMV gH/gL complex model in ribbon representation showing the interaction sites with the neutralizing antibodies 13H11 (yellow circle) and 3G16 (orange circle), this study, and the previously identified MSL-109 binding site (green circle) [28].

3G16 Fab binding induced significant reduction of deuterium uptake in two gH peptides, 677–684 and 705–725 (Fig 3A), demonstrating that 3G16 binds to a composite, not linear, epitope. In addition, we were able to narrow down the second peptide to the unique amino acid sequence 705–708 since two additional gH overlapping peptides, 709–725 and 711–725, presented the same deuterium incorporation in the presence or absence of 3G16 Fab (Fig 3A).

Only the gH peptide 238–247 showed a reduction in deuterium uptake in presence of the 13H11 Fab (Fig 3B). Although the difference is of low amplitude, it was highly reproducible in three independent experiments. A similar analysis was previously performed with the gH/gL/MSL-109 complex, where the peptides 403–419 and 442–446 showed a reduction in deuterium uptake and were identified to be part of the MSL-109 epitope ([28]; Fig 3C).

Overall the HDX-MS results were consistent with the EM analysis in identifying peptides in the C-terminal domain of gH for gH/gL/3G16 and the central region of gH for gH/gL/13H11, respectively (Fig 3C). In addition, the HDX-MS data together with the competition and EM data suggest that the two Fabs compete via their constant domains but not via binding to the same epitope (Fig 3).

In summary, the combination of EM and HDX-MS identified two new neutralizing epitopes in gH/gL.

### Localization of Pentamer specific neutralizing antibodies binding sites

**Fig 4:**
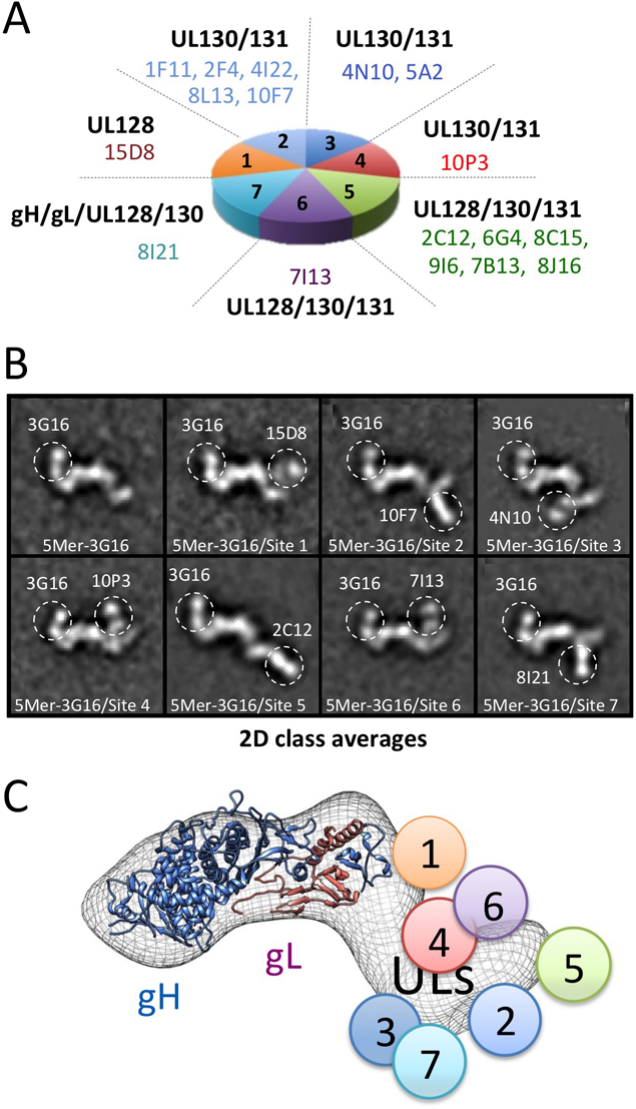
Pentamer specific neutralizing antibodies epitope mapping by NS 2D EM and competition studies. **(A)** Pentamer-specific human neutralizing antibodies including those characterized in this study as classified in [29]. Each slice in the pie represents a different group of antibodies. **(B)** Reference free 2D EM analysis reveals the position of each binding site on the Pentamer for each group as shown in (A). From our EM analysis, Site 1, Site 4 and Site 6 antibodies bind to the upper portion of the Pentamer with Site 4 and Site 6 binding to a very similar position. Site 5 antibodies bind at the tip of the ULs protrusion, while Site 2, Site 3 and Site 7 antibodies bind on the lower portion of the Pentamer. **(C)** Schematic representation of the site of interaction between Pentamer and neutralizing antibodies described in this study as inferred from EM and competition studies.

We then focused our attention on Pentamer-specific neutralizing antibodies previously isolated from the memory B-cell repertoire of HCMV immune donors [29]. These antibodies were previously assigned to seven distinct sites based on binding and cross-competition experiments on cells transfected with different combinations of the gH, gL, UL128, UL130, UL131A genes [29] (Fig 4A). Importantly, this study identified subunits of the Pentamer that are required for binding to antibodies belonging to each of these groups. To identify more precisely the location of the epitopes of these antibodies on the intact complex we used negative stain EM analysis. We reconstituted a Pentamer/3G16 complex bound to additional individual Fabs representing each site and analyzed them by EM and single particle analysis (Fig 4B). In preliminary experiments, 3G16 was found to improve the overall quality of the EM images of the Pentamer/Fabs complexes likely by decreasing flexibly of the gH C-terminal domain.

We started our analysis with 15D8 (Site 1), which has been shown to cross-react specifically with UL128 [29] (Fig 4A). Reference free 2D analysis of Pentamer/3G16/15D8 showed that the 15D8 Fab binds to a region contiguous to the N-terminal portion of the gH/gL complex (Fig 4B), consistent with the proposed proximity between UL128 and gL [28]. Analysis of Pentamer/3G16 bound to 10F7 (Site 2), specific for UL130/UL131A, indicated that this Fab recognizes a distal region of the Pentamer forming a separated domain protruding from gH/gL (Fig 4A and 4B). We used 4N10, also reported by Macagno and colleagues [29] to cross-react with UL130/UL131A, as a representative Fab for Site 3 (Fig 4A). EM analysis showed that 4N10 binding occurs on a position very close to Site 2 but the Fab is oriented at a very different angle (Fig 4B). Binding of 10P3 (Site 4), specific for UL130/UL131A, occurs on the side opposite to Sites 2 and 3 and close to Site 1 (Fig 4A and 4B). 2C12 (Site 5), specific for UL128/UL130/UL131A, was bound to the most distal portion of the Pentamer from the gH/gL region whereas 7I13 (Site 6), an antibody specific for UL128/UL130/UL131A, bound to a similar position as Site 4 (Fig 4A and 4B). Finally, analysis of 8I21 (Site 7), specific for gH/gL/UL128/UL130, showed a very similar localization described for Sites 2 and 3 (Fig 4A and 4B).

Together, our EM analysis identifies two major surfaces on UL128/UL130/UL131A as targets for neutralizing antibody binding. One surface on one side of the Pentamer includes Sites 1, 4 and 6, whereas a second one, on the opposite side, includes Sites 2, 3, 5 and 7. Of note with the exception of Site 5 most of the antibodies seem to cluster in a similar area close to the bottom of the V-shaped part of the UL extension (Fig 4B).

### Binding competition studies

**Fig 5:**
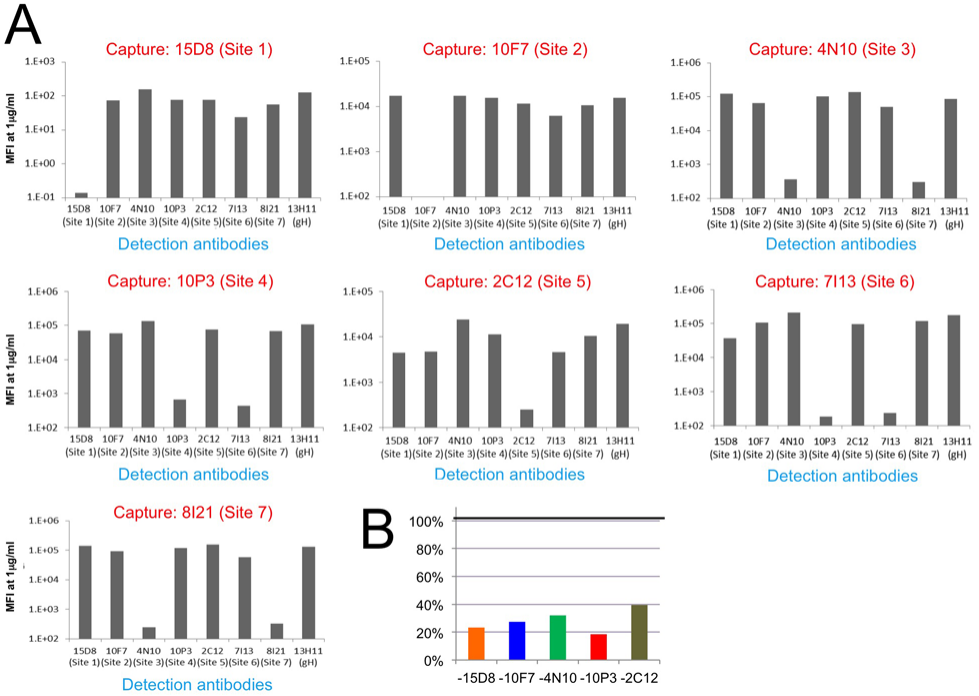
HCMV Pentamer antibody binding competition. HCMV Pentamer antibody binding competition in a Multiplex assay. **(A)** On the x axis are the individual biotinylated detection monoclonal antibodies; on the y axis is the median fluorescence intensity (MFI). Each panel represents the results from one capture antibody coupled to Luminex beads. Because there are no repeated epitopes on the pentameric complex, significant reduction in signal indicates competition between the capturing antibody and the detection antibody. The results indicate that Site 1, 2 and 5-specific monoclonal antibodies only competed with themselves. Site 3 and 7-specific antibodies competed with each other, and the same was observed between Site 4 and 6-specific antibodies. **(B)** Binding of Pentamer to site-specific monoclonal antibodies in the presence of four other non-competing antibodies. Pentamer pre-incubated with pools of four non-competing monoclonal antibodies identified in A was captured with Luminex beads coated with a fifth monoclonal antibody (indicated on the x-axis). The signal for each capture is represented as a percentage of the signal obtained in the absence of added antibody pools (on the y-axis). The five antibodies used in this assay were 15D8 (site 1), 10F7 (site 2), 4N10 (site 3), 10P3 (site 4) and 2C12 (site 5).

To gain additional insights into the location of the different epitopes, we established a Multiplex assay to assess competition for Pentamer binding among the different monoclonal antibodies (Fig 5 and S2 Fig). As suggested by the EM 2D class averages, 10P3 (Site 4) and 7I13 (Site 6), which appear to bind to the same location on the complex, competed for Pentamer binding in Multiplex (Fig 5A). However, Multiplex analysis showed that neither of the Sites 4 nor 6 antibodies cross-compete with 15D8 (Site 1) despite EM showing that their binding sites are in proximity to each other on the Pentamer surface (Fig 4B). Similarly, 10F7 (Site 2) and 4N10 (Site 3) did not show cross-competition in Multiplex (Fig 5A) demonstrating that, despite close spatial proximity (Fig 4B), these epitopes are not actually overlapping. Finally, 8I21 (Site 7), which occupies a very similar localization described for Sites 2 and 3 (Fig 4B), cross-competed with 4N10 (Site 3) but not 10F7 (Site 2) (Fig 5A), indicating that Site 2 and Sites 3–7 are structurally distinct. In summary, the combination of EM and binding studies demonstrates that this panel of monoclonal antibodies binds to five distinct non-competing sites on the Pentamer surface (i.e. Sites 1, 2, 3–7, 4–6, 5; Fig 4C).

Finally we tested if the antibodies that did not compete as pairs could bind to Pentamer at the same time. Pentamer was mixed with antibodies targeting four of the non-competing Pentamer specific sites and competition with an antibody binding to the fifth site was assessed with the Multiplex assay (S3 Fig). Interestingly, this analysis revealed that simultaneous binding of four antibodies interfered with binding of the fifth one, though the magnitude of the effect differed across the five antibodies tested (S3 Fig). The antibodies that bind to these five sites do not compete among each other when tested as pairs (S3 Fig) suggesting that the competition effect observed when multiple antibodies are mixed together with Pentamer may be caused by allosteric effects though steric hindrance effects cannot be excluded completely.

### 3D reconstruction of Pentamer antibody complexes

**Fig 6:**
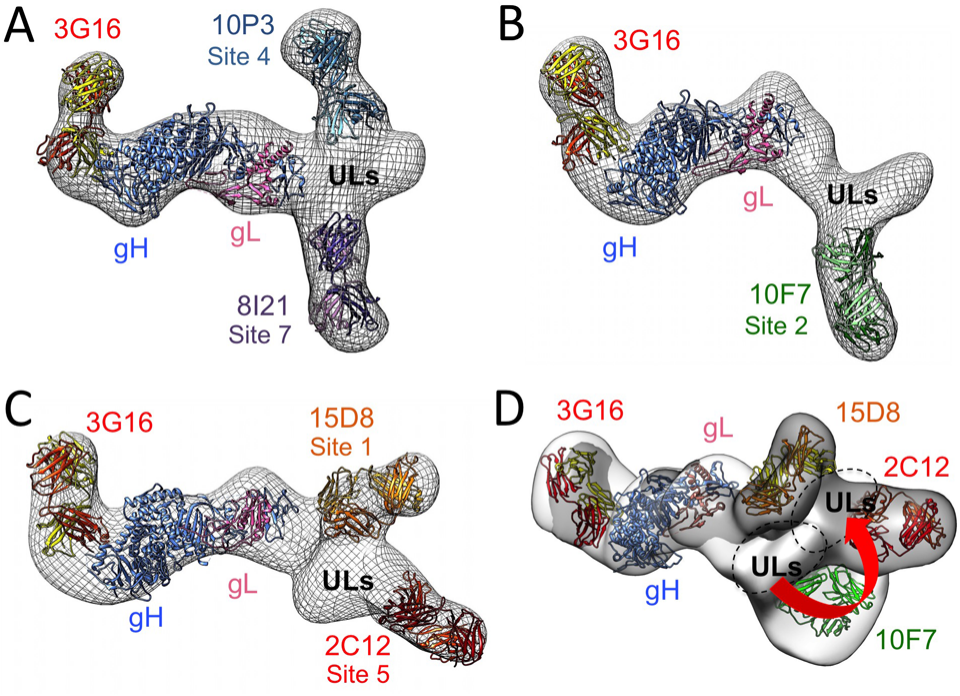
3D Negative Staining EM reconstructions of the HCMV Pentamer bound to neutralizing antibodies. **(A)** RCT reconstruction of Pentamer bound to 10P3, 3G16 and 8I21, **(B)** bound to 10F7 and 3G16 and **(C)** bound to 8I21, 3G16 and 2C12. An HSV based HCMV gH/gL model was fitted with good confidence into the density map comprising gH/gL and the density emerging from the C-terminal region of gH can accommodate a model obtained for the 3G16 Fab. 8I21, 10P3, 10F7, 2C12 and 15D8 Fab models also fit well in the electron densities described by 2D reference free classes and recognize the region of the Pentamer occupied by the ULs proteins. **(D)** Superposition of the gH/gL/3G16 region of the RCT reconstructions of Pentamer/3G16/10F7 (gray) and Pentamer/3G16/15D8/2C12 (white) complexes reveals a movement of the ULs density of roughly 30 degrees.

To gain additional structural insights into the interaction between Pentamer and this set of human neutralizing antibodies we determined RCT 3D-reconstructions of Pentamer bound to the corresponding Fabs. For these reconstructions we selected only Fabs that do not compete among each other for Pentamer binding to define structurally distinct epitopes. Therefore RCTs of Pentamer/3G16/10P3/8I21, Pentamer/3G16/10F7 and Pentamer/3G16/15D8/2C12 complexes at a resolution of respectively ~31 Å, ~30 Å and ~39 Å were obtained. These reconstructions were sufficient to describe the spatial organization of the Pentamer components and define the region of neutralizing antibodies interaction with the ULs (Fig 6A–6C). Also in this case, an HSV-based model of HCMV gH/gL was first fitted into the density maps. Extra densities emerging from the gH C-terminal region and from the UL subunits were consistent with the size of Fabs and each well accommodated a Fab model (Fig 6A–6C). The 2D reconstructions were used to assign the densities protruding from the Pentamer to each of the Fabs. A comparison of the RCT structures of gH/gL/gO and Pentamer revealed a similar architecture of the gH/gL portion bound to 3G16 and confirmed that the gO and UL subunits interact with a common surface on gH/gL (S4 Fig).

Finally, we superimposed the EM 3D reconstructions by manually aligning the surfaces corresponding to their gH/gL/3G16 regions. The Pentamer/3G16/10F7 and Pentamer/3G16/15D8/2C12 reconstructions gave the most reliable superposition. This analysis revealed a good structural conservation of the gH/gL portion of the complex and of its interaction with 3G16. However, this superposition also showed that the ULs are rotated to a different angle relative to gH/gL in the two complexes (Fig 6D). Therefore, antibody binding seems to lock the Pentamer in similar though distinct conformations. The differences in the orientation of the ULs among some of the Pentamer/antibody complexes may in part explain the competition observed when multiple antibodies are mixed simultaneously with Pentamer. Together the EM analysis and the competition data define the location of Pentamer-specific neutralizing antibody binding sites and suggest that the ULs portion of the Pentamer is conformationally flexible.

### Chemical cross-linking identifies Pentamer residues proximal to neutralizing sites

To identify regions of the Pentamer recognized by neutralizing antibodies at the amino acid level, we carried out chemical cross-linking coupled to MS analysis on purified Pentamer and Pentamer/Fab complexes. Disulfosuccinimidyl glutarate (DSSG), a homo-bifunctional cross-linker that reacts with the primary amines of lysines spaced up to 25 Å apart, was used as a cross-linking agent. In preliminary experiments, we observed that UL131A-K27 cross-linked to a large number of lysines on the Pentamer suggesting that this lysine is in a highly flexible part of the molecule. We therefore introduced the UL131A-K27R mutation to simplify the MS analysis and confirmed that this mutation does not affect antibody binding.

**Fig 7:**
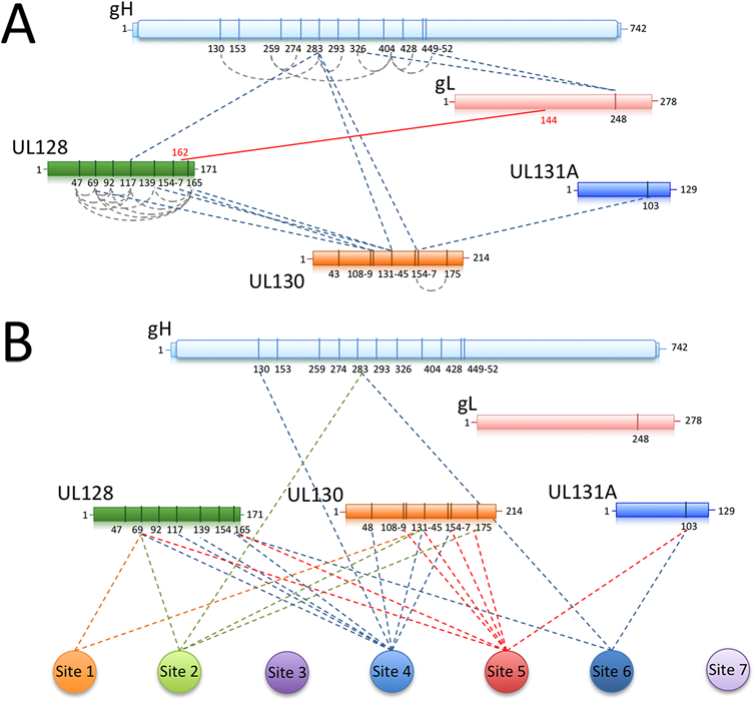
Chemical crosslinking and MS analysis of Pentamer and Pentamer/Fab complexes. Chemical crosslinking coupled to MS was used to identify exposed lysines that are in proximity within the Pentamer complex or between the Pentamer and neutralizing Fabs bound to it. **(A)** UL128 contains many exposed lysines able to crosslink inter-molecularly and intra-molecularly with gH (K283) and UL130 (K108-K131). The central portion of UL130 (K108-K154) crosslinks gH (K283) and UL131 (K103). The disulfide bridge between gL (C144) and UL128 (C162) is also indicated. **(B)** Crosslinking sites between neutralizing antibodies and Pentamer components are indicated. In agreement with the EM data, the majority of the interaction occurs within the UL128 and UL130 subunits.

Cross-linking of unbound Pentamer showed an intricate network of interactions within all the components (S1 Table; Fig 7A). In particular, a region of gH between lysine 130 and 452 contains many sites of inter-molecular cross-linking. Lysine 283 at the N-terminal end of gH cross-linked UL130-K131-145/154-157 and UL128-K117. In turn, several lysines in UL128 cross-linked to a region of UL130 extending from lysine 108 to lysine 131. This 20 amino acid fragment in UL130 is involved in interactions with lysines in gH as well as with UL131A-K103 suggesting a central hub of interaction in the Pentamer. We also observed a large number of lysines in UL128 being internally cross-linked, indicating that this component is exposed on the complex and could potentially represent a target for antibody neutralization. Finally, no lysines were cross-linked in the N-terminus of UL130 suggesting a more buried location in the Pentamer. Taken together the cross-linking data indicate a strong interconnection between the ULs and the N-terminal region of gH/gL confirming our biochemical and EM results.

A similar approach was utilized on Pentamer bound to neutralizing Fabs (S1 Table; Fig 7B). Despite numerous attempts, no cross-linking was observed for Sites 3 and 7 antibodies, suggesting lack of exposed lysines in their proximity. All of the other Fabs cross-linked lysines in UL128 and UL130 (S1 Table) and some also cross-linked a lysine on UL131A, (UL131A-K103). Of note, peptides containing or adjacent to crosslinked lysines in the complexes with Fabs 10P3 (Site 4) (UL128-K165 and UL130-K48), 2C12 and 7I13 (Site 5 and 6 respectively) (UL131A-K103) were previously reported to raise neutralizing antibodies in immunization experiments in mice [41] and could therefore be part of epitopes for additional neutralizing antibodies [31].

Overall the cross-linking data identifies regions of the complex that are exposed and available for antibody binding and suggest that most antibodies examined here bind close to UL128 and UL130. The analysis also identifies a new set of UL subunit peptides that may be potentially used to raise and isolate new HCMV neutralizing monoclonal antibodies or as a component of a HCMV vaccine.

### Antibody binding affinities for gH/gL/gO and Pentamer

The gH/gL- and Pentamer-specific antibodies were tested for binding to purified complexes by SPR. Most of the antibodies bound very tightly to the recombinant proteins with K_D_s (M) between 1.0e^-9^ and 6.0e^-11^ (Table 1). gH/gL specific antibodies bound similarly to recombinant gH/gL/gO and Pentamer confirming that these epitopes are structurally conserved in the two complexes. We also noted that the gH/gL-specific antibodies 3G16 and 13H11 bind to the recombinant complexes equally well although they neutralize infection of epithelial cells at titers 10–100 fold less than Pentamer-specific antibodies [29]. This discrepancy likely reflects differences in the neutralization mechanism between gH/gL- and the UL-specific antibodies.

**Table 1:** Characterization of HCMV neutralizing antibodies. Binding affinities of recombinant Pentamer and gH/gL/gO to human neutralizing antibodies as measured by SPR.

Sample	Site	Antibody	ka (1E+5/Ms)	kd (1E-4/s)	K_D_ (nM)	R_max_ (RU)	Chi^2^ (RU^2^)	Ligand Level (RU)
Pentamer	1	15D8	6.3	6.0	0.95	63.3	0.26	58.6
	2	10F7	7.1	7.5	1.05	23.7	0.114	46.8
	3	4N10	7.9	0.6	0.08	23.2	0.109	12.2
	4	10P3	7.4	0.7	0.09	54	0.333	51.8
	5	2C12	4.2	0.4	0.11	35.2	0.0727	52
	6	7I13	7.9	0.5	0.06	88.6	0.704	48.2
	7	8I21	3.4	0.7	0.19	33.26	0.0817	37.5
	gH-1	3G16	4.3	0.9	0.21	26.9	0.0706	53.2
	gH-2	13H11	3.1	1.9	0.62	11.4	0.0465	72.6
	gH-3	MSL-109*	9.2	0.7	0.08	104.1	0.0254	48
gH/gL/gO	gH-1	3G16	9.6	0.8	0.08	43.9	0.261	55.8
	gH-2	13H11	4.8	1.1	0.24	19.6	0.099	84.5
	gH-3	MSL-109*	10.4	0.7	0.06	90.6	0.0294	45

*MSL-109 values previously reported in Ciferri *et al*., 2015.

## Discussion

Here we have characterized the interaction between gH/gL/gO and Pentamer with a panel of naturally-elicited human neutralizing monoclonal antibodies [29]. Our data confirm the presence of two major regions for neutralizing antibody binding. The first region involves the gH part of the complexes similar to that described for EBV and HSV [42,43]. We show that this region, and its interaction with neutralizing antibodies, is structurally conserved in gH/gL/gO and Pentamer. It is important to note that the gH/gL region is targeted by antibodies neutralizing HCMV entry in all cell types albeit less potently than the Pentamer-specific antibodies in epithelial and endothelial cells [29]. This region may play a role in steps of the viral fusion process, such as gB binding. In this respect we observed that the binding site for 13H11 is in proximity of a site proposed to mediate gB binding in HSV-2 gH/gL [40].

The second region comprises different neutralizing sites on UL128/UL130/UL131A and it likely includes the binding site for the entry receptor for epithelial/endothelial cells. EM analysis and competition studies using purified Pentamer show that the neutralizing sites can be subdivided into two groups defining two distinct surfaces in the UL region of the Pentamer. One surface includes Sites 1, 4, and 6 whereas the second surface includes Sites 2, 3, 5 and 7. These two surfaces are on opposite sides of the elbow formed by the V-shaped ULs component of the Pentamer. It seems unlikely that both surfaces are involved in receptor binding and we speculate that some of the antibodies may instead prevent conformational changes required for activation of membrane fusion. Consistent with this hypothesis comparison of 3D EM analysis of Pentamer/Fab complexes suggest that the ULs portion of the Pentamer is flexible and trapped in different conformations by different Fab combinations. Alternatively, one of the two areas targeted by the neutralizing antibodies may interfere with the interaction of Pentamer with additional cellular and/or viral proteins following receptor binding.

Crosslinking coupled to MS analysis of Pentamer bound to representative neutralizing antibodies for each of the sites on the Pentamer showed that most of them bind in proximity of UL128 and UL130 suggesting that UL131 is partially buried in the complex. Indeed western blot analysis using Cytotect, human IgGs from HCMV positive individuals that contain a high fraction of Pentamer specific neutralizing antibodies, or a polyclonal sera from rabbits immunized with MF59 adjuvanted Pentamer protein fail to detect UL131 in the purified complex (S5 Fig).

Immunization of mice with a subunit Pentamer vaccine adjuvanted with MF59 was shown to raise high titers of antibodies that competed with representative antibodies binding each of the neutralizing sites described above [33,34]. The subunit vaccine raised neutralizing responses in mice that were comparable to or higher than those observed in infected human subjects [33,34]. Therefore, the Pentamer subunit appears to expose all the functionally relevant neutralizing sites including those that are hidden in the context of the native virus particle (e.g. MSL-109 binding site; [37]).

In conclusion, using EM, HDX and chemical cross-linking with MS analysis, we have identified sites on the Pentamer that are targeted by neutralizing antibodies. Together these findings will facilitate the analysis of the human antibody response to HCMV infection and vaccination. Finally, these data will support the development of a new generation of Pentamer-based HCMV vaccines with the potential to elicit potent and clinically relevant protective neutralizing antibody responses.

## Material and Methods

### Generation of HCMV glycoprotein and Fab constructs

Human HCMV Merlin strain gH, gL, gO, UL128, UL130 and UL131A genes synthesized by GeneArt (Regensburg, Germany) codon-modified for expression in *Homo sapiens*, and carrying an optimal Kozak sequence immediately 5’ of each gene, were sub-cloned using NheI/KpnI restriction sites into the pcDNA3.1(-)A plasmid expression vector (Invitrogen, Life Technologies; Carlsbad, CA). The gH gene was terminated at amino acid 715 and therefore was missing the transmembrane domain and cytoplasmic tail. UL130, gO and all the Fab VH chains indicated in the text, and characterized elsewhere [29], were fused to a C-terminal TEV-cleavable Strep-tag II for purification purposes.

### Cell culture, protein purification

293EBNA cells (Invitrogen, Life Technologies; Carlsbad, CA) were transfected with individual plasmids encoding each subunit. Pentamer, gH/gL/gO as well as all the Fabs were purified using the double Strep-tag at the C-terminus of UL130, gO and of the VH chain, respectively. Affinity purified complexes were eluted from the resin using 5 mM desthiobiotin containing elution buffer (25 mM Tris pH 7.5, 300 mM NaCl). The final step of the purification of both HCMV complexes and Fabs consisted of size exclusion chromatography (SEC) on a Superose 6 PC 3.2/30 or Superdex 200/30 column, equilibrated in 25 mM Tris pH 7.5, 300 mM NaCl. Complexes between Fabs and either gH/gL/gO or Pentamer were generated by incubation of these proteins on ice for 2 h using a 1.5-fold molar excess of Fab and purified by SEC to remove Fab excess.

### Native gel-shift assay

Purified gH/gL monomer or dimer protein (previously described in [28]) were incubated with 3G16, MSL-109 or 13H11 Fabs in various combinations at a ratio of 10 μg gH/gL to 5 μg each Fab for 1 h at room temperature (RT) before being resolved on NativePAGE Novex 3–12% Bis-Tris Protein Gels (Invitrogen Inc.) along with NativeMark Unstained Protein Standard (Invitrogen Inc.). The gels were subsequently stained with Coomassie Blue to visualize the bands.

### Chemical cross-linking using isotope labeled cross-linkers and MS identification of cross-linked peptides

HCMV Pentamer or Pentamer/Fab complexes (36 pmol) were mixed with a 600-fold excess of isotope-labeled cross-linker di-(sulfosuccinimidyl)-glutarate (1:1 mixture of light DSSG-d_0_ and heavy DSSG-d_6_) (Creative Molecules, Victoria, BC, Canada) in a final volume of 50 μl of 10 mM HEPES, pH 7.5, 300 mM NaCl at RT. The reaction was stopped after 30 min by adding 5 μl of 1 M ammonium bicarbonate.

Cross-linked proteins were reduced with 5 mM TCEP (tris (2-carboxyethyl) phosphine; Thermo Scientific, Rockford, IL, USA) at 37°C for 30 min and alkylated with 10 mM iodoacetamide (Sigma-Aldrich, St. Louis, MO, USA) for 30 min in the dark. Proteins were first digested with endoproteinase Lys-C (Wako, Neuss, Germany) at an enzyme-to-substrate ratio of 1:100 (w/w) for 3 h at 37°C and subsequently with sequencing grade trypsin (Promega, Madison, WI, USA) at an enzyme-to-substrate ratio of 1:50 (w/w) at 37°C overnight. Peptides were acidified with 2% formic acid (Sigma-Aldrich) and purified by solid-phase extraction (SPE) using C_18_ cartridges (Sep-Pak; Waters, Milford, MA, USA). The SPE eluate was evaporated to dryness and reconstituted in 20 μl of SEC mobile phase (water/acetonitrile/TFA, 70:30:0.1). 15 μl were injected on a GE Healthcare (Uppsala, Sweden) Äkta micro system. Peptides were separated on a Superdex Peptide PC 3.2/30 column (300×3.2 mm) at a flow rate of 50 μl min^−1^ using the SEC mobile phase [44]. Two-minute fractions (100 μl) were collected into 96-well plates.

LC-MS/MS analysis was carried out on an Eksigent 1D-NanoLC-Ultra system connected to a Thermo LTQ Orbitrap XL mass spectrometer equipped with a standard nanoelectrospray source. SEC fractions were reconstituted in mobile phase A (water/acetonitrile/formic acid, 97:3:0.1). A fraction corresponding to an estimated 1 μg of peptides was injected onto a 11 cm × 0.075 mm I.D. column packed in house with Michrom Magic C18 material (3 μm particle size, 200 Å pore size). Peptides were separated at a flow rate of 300 nl min^−1^ ramping a gradient from 5% to 35% mobile phase B (water/acetonitrile/formic acid, 3:97:0.1).

Cross-linked peptides were identified using an in-house version of the dedicated search engine, xQuest [45]. Tandem mass spectra of precursors differing in mass by 6.037660 Da (difference between DSSG-d0 and DSS-d6) were paired if they had a charge state of 3+ to 8+ and were triggered within 2.5 min of each other. These spectra were then searched against a pre-processed *fasta* database containing the target sequences. A valid identification of the cross-linked peptides required an xQuest score of at least 16 (corresponding to a false discovery rate of > 5%) and at least four bond cleavages in total or three in a series for each peptide and a minimum peptide length of six amino acids.

### EM data collection and processing

EM grids were prepared by placing five microliters of purified sample on a freshly glow discharged 400-mesh copper grid covered with a thin layer of continuous carbon. After 30 sec of incubation, the grid was stained with 5 drops of a freshly prepared 2% (w/v) uranyl formate solution. Samples were imaged on a Tecnai Spirit T12 transmission electron microscope operating at 120 keV with a magnification of 49,000× (1.57 Å/pixel at the detector level) using a defocus range of −0.8 to −1.2 μm. Images were recorded on a Gatan 4096 × 4096 pixel CCD camera under low-dose conditions. Random Conical Tilt (RCT) dataset images were collected at −56° and 0°. Particle picking for all datasets was executed using the Eman2 e2boxer software [46] and a 224 × 224-pixel particle box size window. All datasets were band-pass filtered with a 20-Å low-pass—200-Å high-pass cutoff. Reference Free 2D class averaging of individual complexes was generated using iterative Multivariate Statistical Analysis (MSA) and Multi-Reference Alignment (MRA) in IMAGIC [47] including, on average, ~20 particles per class average. Epitope localization was performed by alignment and cross correlation between reference free 2D classes of Fab bound and unbound samples and by subtracting the unbound class averages from the Fab-bound classes. The obtained difference maps, considered meaningful only if the signal was at least three standard deviations above the mean, indicated the location of each Fab on HCMV complexes. RCT three-dimensional models of the negatively-stained gH/gL/gO and Pentamer complexes were generated by collecting 50 tilt-pair images (0° and 56°) using the same conditions described above. Pair tilts were manually selected for a total of approximately 5000 particle pairs for each sample. The ML2D in the XMIPP package was used to generate reference-free 2D averages from the 0° micrographs [48]. 3D RCT reconstructions were finally generated using SPIDER routines integrated into Appion starting from the more populated RCT classes.

### HDX-MS analysis of gH/gL/Fab complexes

gH/gL/Fab complexes were formed by incubating for 30 min at RT ~300 pmoles of gH/gL-C144S to 1:2 molar excess of 3G16 and 13H11 Fabs. To perform sample labeling, deuterated buffer (Tris-HCl 25mM, NaCl 150 mM, pH 7.1) was added at RT, reaching a deuterium excess of 78%. At different time points (between 30 sec and 30 min), 30 μL of sample were removed and mixed with an equal volume of ice-cold 200 mM sodium phosphate, 4 M guanidinium chloride, 200 mM TCEP, pH 2.1 buffer to quench the deuterium exchange reaction and promote Fab dissociation. Quenched aliquots were flash frozen in liquid nitrogen and stored at -80°C before analysis. Unbound gH/gL-C144S was used as control. Samples were analyzed using a Waters nano-ACQUITY UPLC with HDX Technology coupled to a Waters SynaptG2 mass spectrometer equipped with a standard ESI source (Waters). The data generated with this equipment was analyzed and interpreted using a previously reported method [28]. Only peptides present in at least three repeated digestions were considered for the analysis.

### Binding studies by Surface Plasmon Resonance (SPR)

SPR single cycle kinetics experiments were carried out on a Biacore T100 instrument using a human IgG binder kit as previously described [28]. Both ligand and analyte samples were diluted in HBS-EP buffer (GE healthcare BR100669). One channel of a CM5 chip was used as reference while the second was used to capture HCMV neutralizing IgG. Ligand levels were maintained between 45–85 RU. Concentrations of Pentamer or gH/gL/gO from 0, 3.125, 6.25, 12.5 and 25 nM were injected over the two channels for 120 s at 50 μl/min followed by 600 s of dissociation time. The single cycle kinetic curves were fitted using a 1:1 binding stoichiometry for *k*_*a*_, *k*_*d*_, K_D_.

### Monoclonal antibody competition by ELISA

A capture ELISA assay was performed to determine competing binding of the gH-specific monoclonal antibodies. Individual antibodies were biotinylated using a commercial kit according to manufacturer instructions (NHS-PEG4-Biotin, No-Weigh Format, Thermo Scientific, cat# 21329). 96-well ELISA plates (Immuno F96 MaxiSorp, Nunc cat# 439454) were coated with 100 μl/well of individual monoclonal antibodies diluted to 1 μg/ml in PBS. Following overnight incubation at 4°C, wells were washed 3 times with 300 μl/well of PBS containing 0.05% (w/v) Tween 20 (wash buffer). The wells were blocked with 100 μl/well of 1% (w/v) BSA in PBS (blocking buffer) for 1 h at RT. The blocking buffer was removed by aspiration and purified gH/gL complex, 200 ng/well, was added to the plates in 100 μl/well of PBS containing 1% (w/v) BSA and 0.1% (w/v) Triton X-100 (sample buffer) and incubated for 1 h at RT. Wells were washed 3 times with 300 μl/well of wash buffer and incubated for 1 h at RT with biotinylated detection monoclonal antibodies in sample buffer, 100 μl/well. After washing, HRP conjugated avidin (Vector cat# A-2004), was added at 100 μl/well of sample buffer and incubated for 1 h at RT. Wells were washed and incubated for 30 min with 100 μl/well of TMB substrate (Rockland cat# TMBE-1000). Following incubation, the reaction was stopped by adding 100 μl/well of 2.0 N Sulfuric Acid (BDH cat# BDH3500). The optical density was determined spectrophotometrically at 450 nm wavelength using a microplate reader (Infinite M200 NanoQuant, Tecan).

### Monoclonal antibody competition by multiplex assay

A capture multiplex assay was performed to determine competing binding of the Pentamer-specific monoclonal antibodies. For direct competition between pairs of monoclonal antibodies Luminex microspheres (MagPlex microspheres, Luminex Corp. cat# MC100XX), of different classification, were coupled with individual monoclonal antibodies by chemical coupling according to manufacturer instructions. Individual antibodies were also biotinylated using a commercial kit (NHS-PEG4-Biotin, No-Weig Format, Thermo Scientific, cat# 21329). In 96 well white plates, 1000 microspheres/well for each classification/monoclonal were mixed in 50 μl/well of DPBS + 1% BSA + 0.05% sodium azide (assay buffer) with purified Pentamer, starting at 100 ng/well with three-fold dilutions down to 0.05 ng/well. After washing three times with 200 μl/well of PBS containing 0.05% (w/v) Tween 20 (wash buffer) to remove excess antigen, individual biotinylated monoclonal antibodies (at 0.75–1.5 μg/ml) were added in separate wells in 50 μl/well of assay buffer and incubated for 1 h at RT with orbital shaking in the dark. After washing, R-Phycoerythrin conjugated Streptavidin (Jackson ImmunoResearch, cat# 016-110-084) was added, 50 μl/well in assay buffer, and incubated for 1 h at RT. After a final wash, Fluorescence intensity was measured using a Luminex FlexMap 3D (Life Technologies model FM3D000).

### Multi-antibodies competition experiment

For evaluation of whether antibodies that did not compete as pairs could bind to Pentamer at the same time, Luminex microspheres were coupled with individual monoclonal antibodies for sites 1 to 5 of the Pentamer (15D8, 10F7, 4N10, 10P3, 2C212). In 96 well white plates, serial dilutions of monoclonal antibody pools containing 1000 ng/well of combinations of four of the five antibodies were made. To these dilutions of antibody pools, 1000 microspheres/well for each classification/monoclonal were added in 50 μl/well of DPBS + 1% BSA + 0.05% sodium azide (assay buffer) together with purified Pentamer, 33 ng/well, and incubated for 1 h at RT with orbital shaking in the dark. After washing three times with 200 μl/well of PBS containing 0.05% Tween 20 (wash buffer) to remove excess antigen, a commercially available biotin conjugated anti His-tag mAb (Rockland, Cat # 200-306-382) for direct detection of the Pentamer, was added with 50 μl/well of assay buffer and incubated for 1 h at RT with orbital shaking in the dark. After washing, R-Phycoerythrin conjugated Streptavidin (Jackson ImmunoResearch, cat# 016-110-084) was added, 50 μl/well in assay buffer, and incubated for 1 h at RT. After a final wash, Fluorescence intensity was measured using a Luminex FlexMap 3D (Life Technologies model FM3D000).

## Supporting Information

S1 Fig gH/gL binding by gH specific monoclonal antibodies. Native gel-shift assay to study binding competition between 3G16, MSL-109 and 13H11 Fabs for **(A)** gHgL homodimer and **(B)** gH/gL-C144S monomer. The shit of the gH/gL bands reveals that 13H11 can bind gH/gL at the same time as either 3G16 or MSL-109. Binding of 3G16 and MSL-109 Fabs appears to be mutually exclusive.
(TIF)

S2 Fig Competition between site-specific monoclonal antibodies for binding to Pentamer. Titration curves from Multiplex experiments outlined in Fig 5A. Each panel (A-G) represents the Luminex signal from a dilution of Pentamer protein ranging from 1 μg/mL to 0.5 ng/mL bound to each of seven different monoclonal antibodies (15D8, 10F7, 4N10, 10P3, 2C12, 7I13 and 8I21) following detection with a particular biotinylated monoclonal antibody (panel A, 15D8; B, 10F7; C, 4N10; D, 10P3; E, 2C12; F, 7I13 and G, 8I21). Antibodies that compete give no detectable signal even at the highest tested concentration of Pentamer, i.e. 4N10 and 8I21 in panels C and G; and 10P3 and 7I13 in panels D and F.
(TIF)

S3 Fig Multiple antibody competition assay. Titration curves for Multiplex experiments outlined in Fig 5B. Each panel represents the Luminex signal from one biotinylated detection antibody (panel A, 15D8; B, 10F7; C, 4N10; D, 10P3; E, 2C12) following pre-incubation of Pentamer with combinations of monoclonal antibodies. “All” represents pre-incubation with all five antibodies (15D8, 10F7, 4N10, 10P3 and 2C12), while “None” represents the signal when no monoclonal antibody was used in pre-incubation. Combinations of four antibodies are indicated in the legend based on the missing antibody in each combination (e.g. “-15D8” represents pre-incubation with 10F7, 4N10, 10P3 and 2C12 etc.). The antibody concentrations ranged from 3 ng/mL to 6 μg/mL. Curves are color-coded as in Fig 5B.
(TIF)

S4 Fig Comparison between RCT structures of gH/gL/gO and Pentamer. 3D-reconstruction of Pentamer bound to 3G16, 8I21 and 10P3 and gH/gL/gO bound to 3G16 were determined by EM using the RCT method. gH/gL subunit bound to 3G16 Fab maintains a similar architecture in gH/gL/gO and Pentamer. Additional densities emerging from the N-terminal region of gH/gL (in red) describe the localization and architecture of gO and ULs.
(TIF)

S5 Fig Recognition of Pentamer by a rabbit polyclonal anti-Pentamer antibody. **(A)** Coomassie-stained SDS-PAGE and **(B)**, anti-Pentamer western blot of purified HCMV Pentamer under boiled and reduced (+/+) or non-boiled and non-reduced (-/-) conditions. Under -/- conditions, the anti-Pentamer polyclonal antibody reveals two distinct bands corresponding to gH/gL/UL128 and UL130. Under +/+ conditions, the antibody reveals four distinct bands corresponding to gH, UL130, gL and UL128. The UL131A band is visible in Coomassie staining under -/- conditions and co-migrates with UL128 under +/+ conditions. All individual bands except UL131A could be verified using N-terminal sequencing. Protein was loaded at 1 μg/well for Coomassie staining and 0.1 μg/well for western blot. The secondary antibody was a goat anti-rabbit IgG IRDye 800CW from LiCor.
(TIF)

S1 Table Chemical Crosslinking coupled to MS of Pentamer bound to neutralizing antibodies. Internal cross-linking (in green) as well as cross-linking between different Pentamer components and Fabs (orange) are reported. For each cross-linking, the subunits involved as well as the amino acid being cross-linked and peptide sequences detected by MS are indicated.
(PDF)
