# Supplementary figures and images for: Antigenic Characterization of the HCMV gH/gL/gO and Pentamer Cell Entry Complexes Reveals Binding Sites for Potently Neutralizing Human Antibodies
Source: PLoS Pathog. 2015 Oct 20;11(10):e1005230. doi: 10.1371/journal.ppat.1005230 (PMC4617720; doi:10.1371/journal.ppat.1005230)

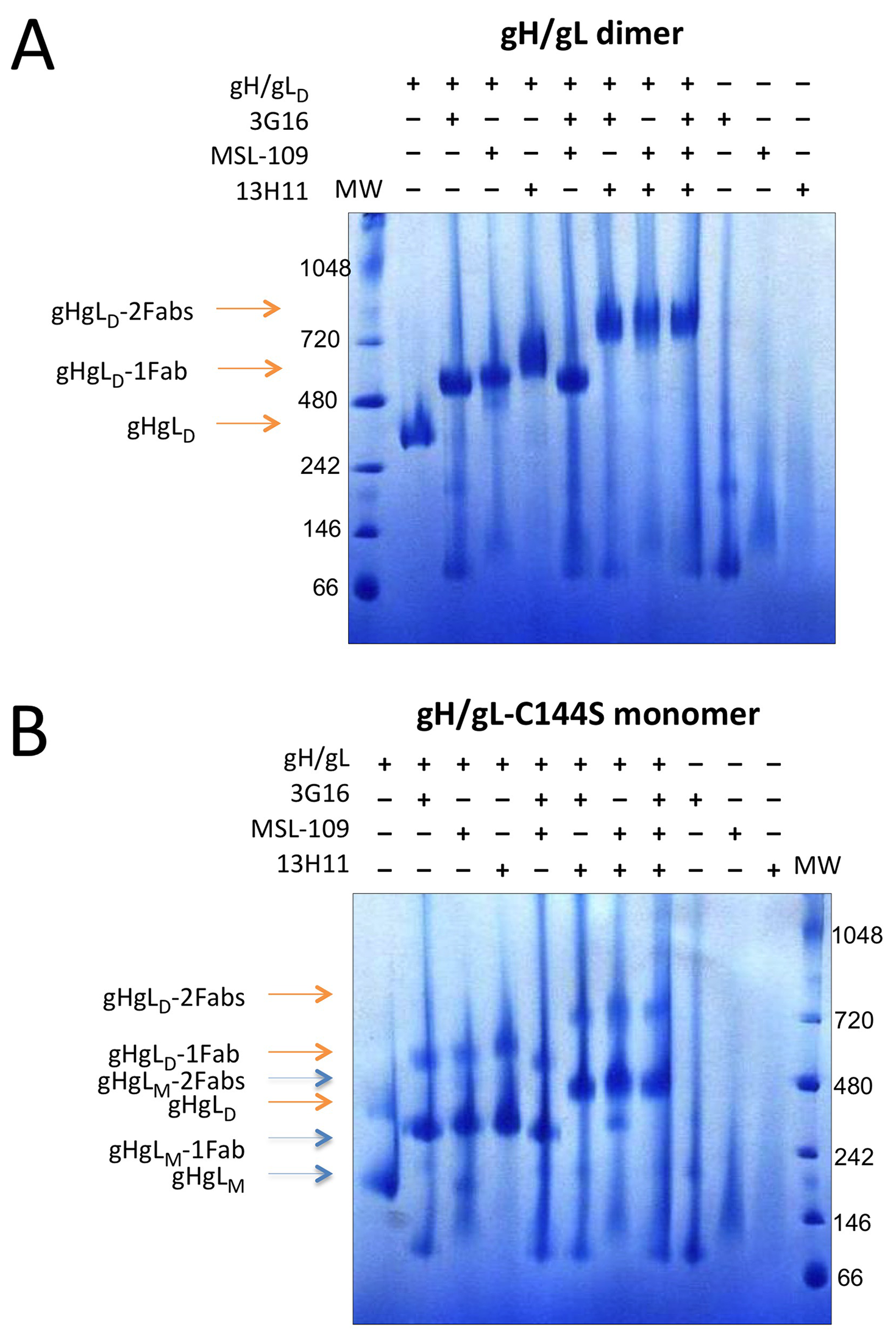

Supplement: S1 Fig — Native gel-shift assay to study binding competition between 3G16, MSL-109 and 13H11 Fabs for (A) gHgL homodimer and (B) gH/gL-C144S monomer. The shit of the gH/gL bands reveals that 13H11 can bind gH/gL at the same time as either 3G16 or MSL-109. Binding of 3G16 and MSL-109 Fabs appears to be mutually exclusive. (TIF) [file ppat.1005230.s001.tif]

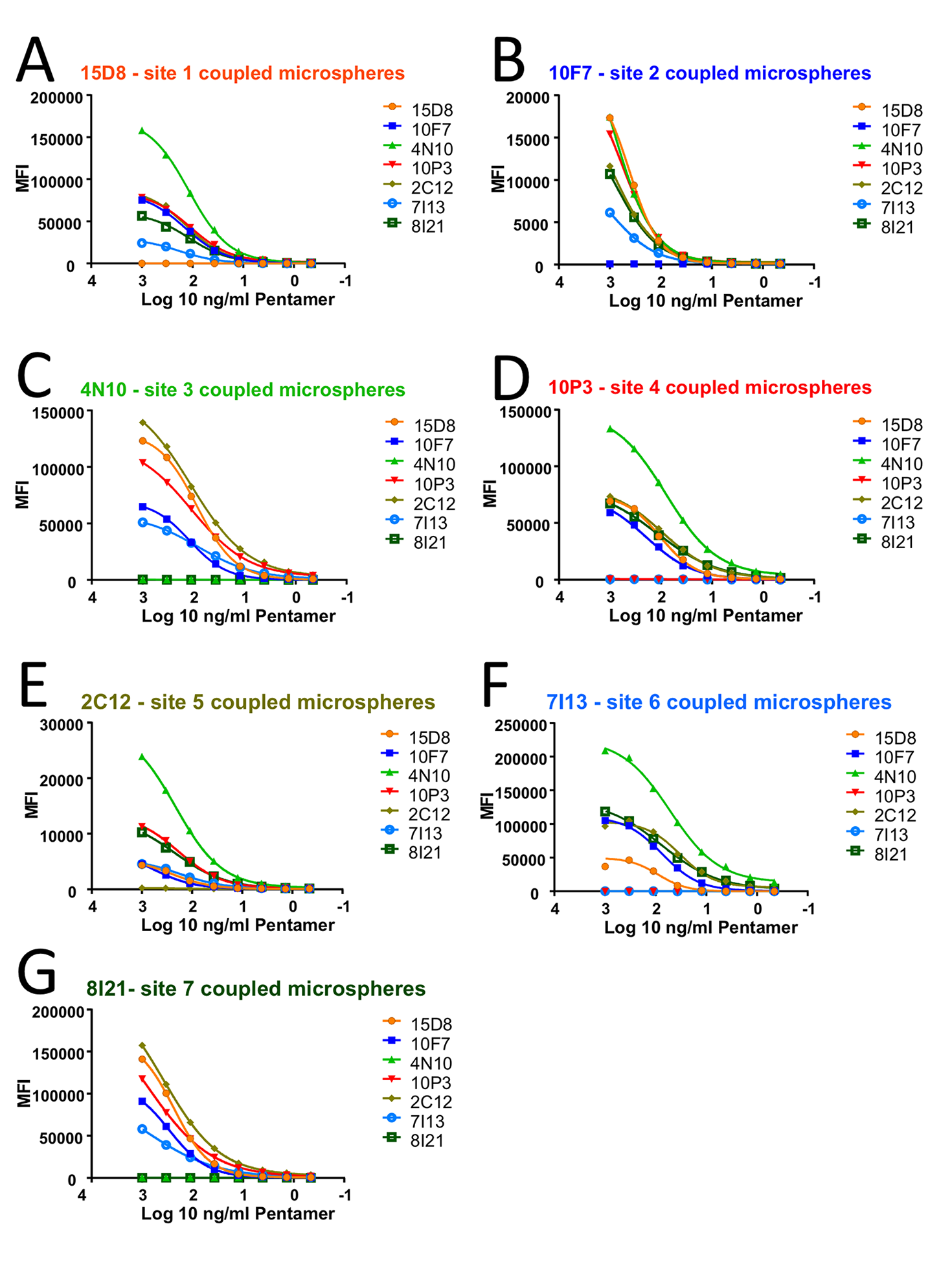

Supplement: S2 Fig — Titration curves from Multiplex experiments outlined in Fig 5A. Each panel (A-G) represents the Luminex signal from a dilution of Pentamer protein ranging from 1 μg/mL to 0.5 ng/mL bound to each of seven different monoclonal antibodies (15D8, 10F7, 4N10, 10P3, 2C12, 7I13 and 8I21) following detection with a particular biotinylated monoclonal antibody (panel A, 15D8; B, 10F7; C, 4N10; D, 10P3; E, 2C12; F, 7I13 and G, 8I21). Antibodies that compete give no detectable signal even at the highest tested concentration of Pentamer, i.e. 4N10 and 8I21 in panels C and G; and 10P3 and 7I13 in panels D and F. (TIF) [file ppat.1005230.s002.tif]

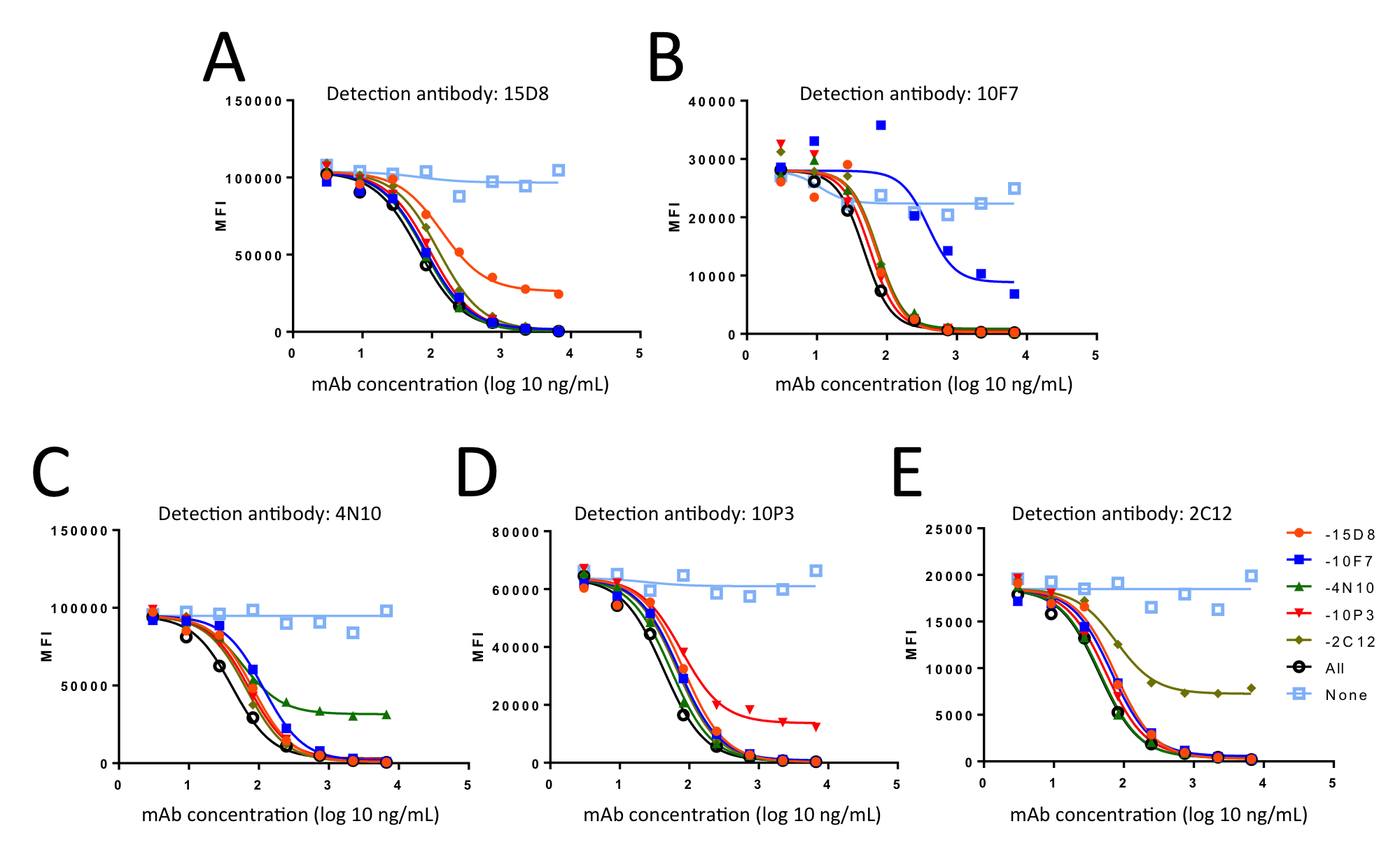

Supplement: S3 Fig — Titration curves for Multiplex experiments outlined in Fig 5B. Each panel represents the Luminex signal from one biotinylated detection antibody (panel A, 15D8; B, 10F7; C, 4N10; D, 10P3; E, 2C12) following pre-incubation of Pentamer with combinations of monoclonal antibodies. “All” represents pre-incubation with all five antibodies (15D8, 10F7, 4N10, 10P3 and 2C12), while “None” represents the signal when no monoclonal antibody was used in pre-incubation. Combinations of four antibodies are indicated in the legend based on the missing antibody in each combination (e.g. “-15D8” represents pre-incubation with 10F7, 4N10, 10P3 and 2C12 etc.). The antibody concentrations ranged from 3 ng/mL to 6 μg/mL. Curves are color-coded as in Fig 5B. (TIF) [file ppat.1005230.s003.tif]

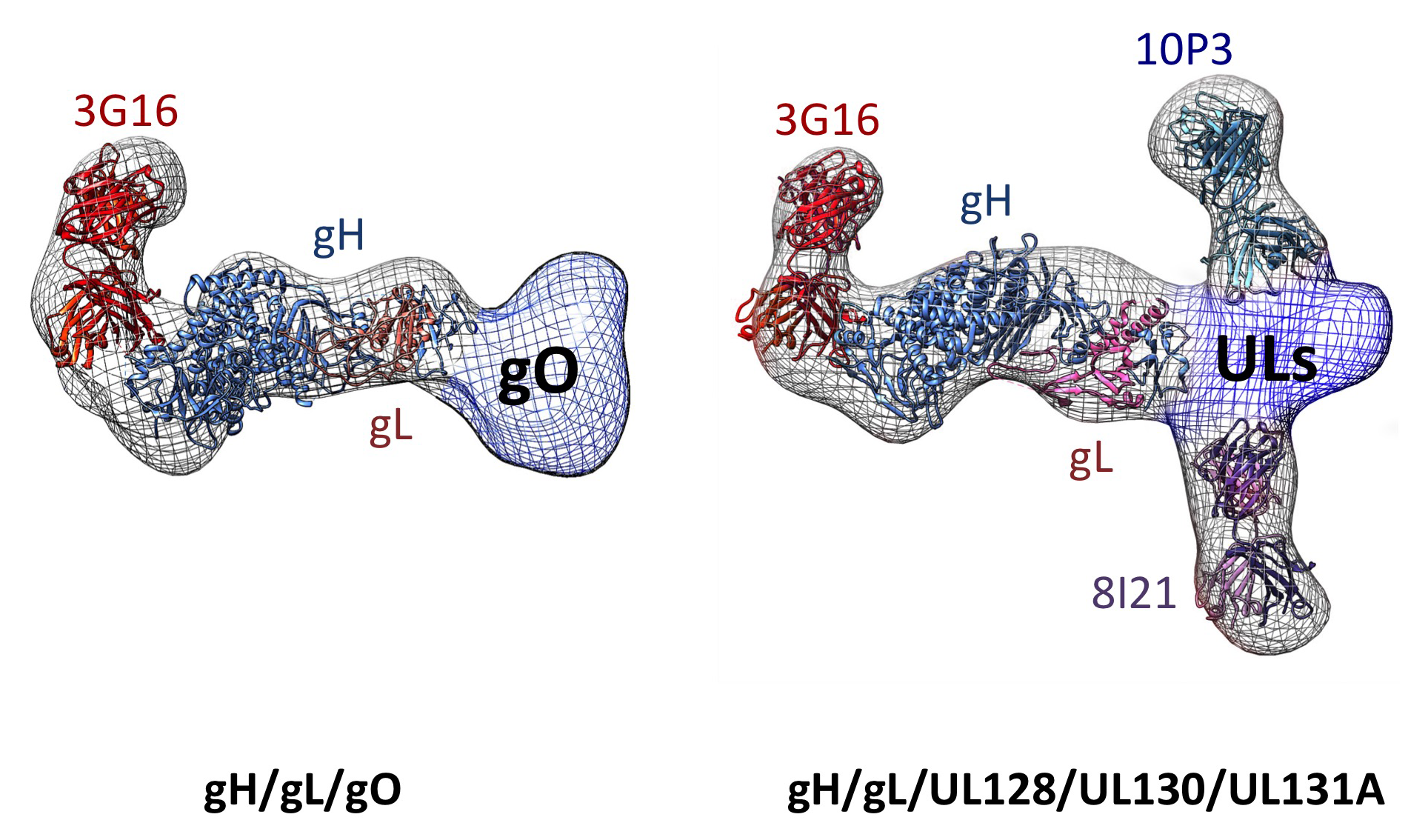

Supplement: S4 Fig — 3D-reconstruction of Pentamer bound to 3G16, 8I21 and 10P3 and gH/gL/gO bound to 3G16 were determined by EM using the RCT method. gH/gL subunit bound to 3G16 Fab maintains a similar architecture in gH/gL/gO and Pentamer. Additional densities emerging from the N-terminal region of gH/gL (in red) describe the localization and architecture of gO and ULs. (TIF) [file ppat.1005230.s004.tif]

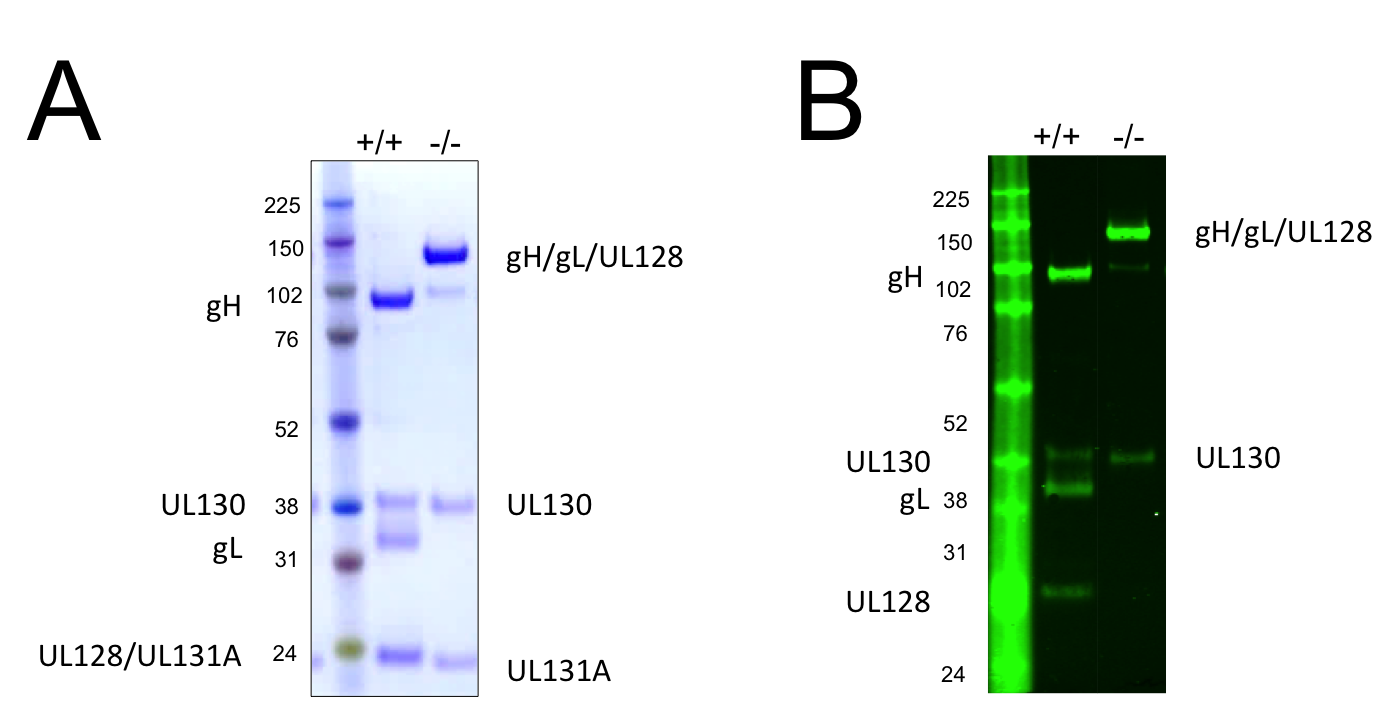

Supplement: S5 Fig — (A) Coomassie-stained SDS-PAGE and (B), anti-Pentamer western blot of purified HCMV Pentamer under boiled and reduced (+/+) or non-boiled and non-reduced (-/-) conditions. Under -/- conditions, the anti-Pentamer polyclonal antibody reveals two distinct bands corresponding to gH/gL/UL128 and UL130. Under +/+ conditions, the antibody reveals four distinct bands corresponding to gH, UL130, gL and UL128. The UL131A band is visible in Coomassie staining under -/- conditions and co-migrates with UL128 under +/+ conditions. All individual bands except UL131A could be verified using N-terminal sequencing. Protein was loaded at 1 μg/well for Coomassie staining and 0.1 μg/well for western blot. The secondary antibody was a goat anti-rabbit IgG IRDye 800CW from LiCor. (TIF) [file ppat.1005230.s005.tif]
