# Supplementary material for: Antigenic Characterization of the HCMV gH/gL/gO and Pentamer Cell Entry Complexes Reveals Binding Sites for Potently Neutralizing Human Antibodies
Source: PLoS Pathog. 2015 Oct 20;11(10):e1005230. doi: 10.1371/journal.ppat.1005230 (PMC4617720; doi:10.1371/journal.ppat.1005230)
Supplement: S1 Table — Internal cross-linking (in green) as well as cross-linking between different Pentamer components and Fabs (orange) are reported. For each cross-linking, the subunits involved as well as the amino acid being cross-linked and peptide sequences detected by MS are indicated. (PDF) [file ppat.1005230.s006.pdf]

Supplementary Table S1

Unbound

Id	Protein1	Protein2	XLType	AbsPos1	AbsPos2	deltaAA	ID score
LNTYALVSKDLASYR-KDQLNR	gH	gH	intra-protein xl	130	283	153	
VLFKAPYQR-QTEKHELLVLVK	gH	gH	intra-protein xl	259	274	15	
VLFKAPYQR-TTLLLYPTAVDLAKR	gH	gH	intra-protein xl	259	404	145	
KDQLNR-HSYLKDPDFLDAALDFNYLDLSALLR	gH	gH	intra-protein xl	283	293	10	
YAVDVLKSGR-TTLLLYPTAVDLAKR	gH	gH	intra-protein xl	326	404	78	
TTLLLYPTAVDLAKR-LVYILSKQNQQHLIPQWALR	gH	gH	intra-protein xl	404	428	24	
TTLLLYPTAVDLAKR-QIADFALKLHK	gH	gH	intra-protein xl	404	449-52	45-48	
CPDGEVCYSPEKTAEIR-CYDFKMCNR	UL128	UL128	intra-protein xl	69	47	22	
QVVHNKLTSCNYNPLYLEADGR-CYDFKMCNR	UL128	UL128	intra-protein xl	92	47	45	
CPDGEVCYSPEKTAEIR-QVVHNKLTSCNYNPLYLEADGR	UL128	UL128	intra-protein xl	69	92	23	
QVVHNKLTSCNYNPLYLEADGR-VNDKAQYLLGAAGSVPYR	UL128	UL128	intra-protein xl	92	117	25	
CYDFKMCNR-VNDKAQYLLGAAGSVPYR	UL128	UL128	intra-protein xl	47	117	70	
CYDFKMCNR-WINLEYDKITR	UL128	UL128	intra-protein xl	47	139	92	
CYDFKMCNR-AKMGYMLQ	UL128	UL128	intra-protein xl	47	165	118	
WINLEYDKITR-AKMGYMLQ	UL128	UL128	intra-protein xl	139	165	26	
CPDGEVCYSPEKTAEIR-VNDKAQYLLGAAGSVPYR	UL128	UL128	intra-protein xl	69	117	48	
CPDGEVCYSPEKTAEIR-WINLEYDKITR	UL128	UL128	intra-protein xl	69	139	70	
CPDGEVCYSPEKTAEIR-AKMGYMLQ	UL128	UL128	intra-protein xl	69	165	96	
QVVHNKLTSCNYNPLYLEADGR-AKMGYMLQ	UL128	UL128	intra-protein xl	92	165	73	
VNDKAQYLLGAAGSVPYR-AKMGYMLQ	UL128	UL128	intra-protein xl	117	165	48	
YQMCVMKLESWAHVFR-IFGAHMVPKQTK	UL130	UL130	intra-protein xl	175	154-7	18-21	

Id	Protein1	Protein2	XLType	AbsPos1	AbsPos2	ID score
IFGAHMVPKQTK-RGGTNKR	UL130	UL131A	inter-protein xl	154-7	103	
YAVDVLKSGR-HLDKYYAGLPPELK	gH	gL	inter-protein xl	326	248	
QIADFALKLHK-HLDKYYAGLPPELK	gH	gL	inter-protein xl	449-52	248	
IFGAHMVPKQTK-KDQLNR	UL130	gH	inter-protein xl	154-7	283	
TASKPSDGNVQISVEDAK-KDQLNR	UL130	gH	inter-protein xl	131-45	283	
VNDKAQYLLGAAGSVPYR-KDQLNR	UL128	gH	inter-protein xl	117	283	
TASKPSDGNVQISVEDAK-WINLEYDKITR	UL130	UL128	inter-protein xl	131-45	139	
TASKPSDGNVQISVEDAK-AKMGYMLQ	UL130	UL128	inter-protein xl	131-45	165	
SSTWVKK-CPDGEVCYSPEKTAEIR	UL130	UL128	inter-protein xl	108-9	69	
SSTWVKK-KKHKR	UL130	UL128	inter-protein xl	108-9	154-7	

Site1

Id	Protein1	Protein2	XLType	AbsPos1	AbsPos2	deltaAA	ID score
LNTYALVSKDLASYR-KDQLNR	gH	gH	intra-protein xl	130	283	153	28.73
TASKPSDGNVQISVEDAK-SSTWVKK	UL130	UL130	intra-protein xl	131-45	108-9	22-37	23.65
YQMCVMKLESWAHVFR-IFGAHMVPKQTK	UL130	UL130	intra-protein xl	175	154-7	18-21	23.44
CPDGEVCYSPEKTAEIR-CYDFKMCNR	UL128	UL128	intra-protein xl	69	47	22	23.31
QTEKHELLVLVK-KDQLNR	gH	gH	intra-protein xl	274	283	9	23.27
VNDKAQYLLGAAGSVPYR-CPDGEVCYSPEKTAEIR	UL128	UL128	intra-protein xl	117	69	48	22.65
GPSVFPLAPSSKSTSGGTAAPGCLVK-RVEPKSCDK	15D8 hc	15D8 hc	intra-protein xl	135	220	85	18.7
QVVHNKLTSCNYNPLYLEADGR-CYDFKMCNR	UL128	UL128	intra-protein xl	92	47	45	18.65
TTLLLYPTAVDLAKR-QIADFALKLHK	gH	gH	intra-protein xl	404	449	45	16.1

Id	Protein1	Protein2	XLType	AbsPos1	AbsPos2	ID score
TASKPSDGNVQISVEDAK-WINLEYDKITR	UL130	UL128	inter-protein xl	131-45	139	24.94
TASKPSDGNVQISVEDAK-RGGTNKR	UL130	UL131A	inter-protein xl	131-45	103	24.08
SYSCQVTHEGSTVEKTVAPTECS-RVEPKSCDK	15D8 lc	15D8 hc	inter-protein xl	204	220	21.09
ADSSPVKAGVETTTPSK-CPDGEVCYSPEKTAEIR	15D8 lc	UL128	inter-protein xl	156	69	20.88
CPDGEVCYSPEKTAEIR-RGGTNKR	UL128	UL131A	inter-protein xl	69	103	19.77
TASKPSDGNVQISVEDAK-ADSSPVKAGVETTTPSK	UL130	15D8 lc	inter-protein xl	131-45	156	19.51
QSNNKYAASSYLSLTPEQWK-CPDGEVCYSPEKTAEIR	15D8 lc	UL128	inter-protein xl	171	69	16.59

Site 2

Id	Protein1	Protein2	XLType	AbsPos1	AbsPos2	deltaAA	ID score
LNTYALVSKDLASYR-KDQLNR	gH	gH	intra-protein xl	130	283	153	26.17
WINLEYDKITR-AKMGYMLQ	UL128	UL128	intra-protein xl	139	165	26	24.56
TTLLLYPTAVDLAKR-YAVDVLKSGR	gH	gH	intra-protein xl	404	326	78	24.27
CPDGEVCYSPEKTAEIR-CYDFKMCNR	UL128	UL128	intra-protein xl	69	47	22	23.31
TASKPSDGNVQISVEDAK-SSTWVKK	UL130	UL130	intra-protein xl	131-45	108-9	22-37	23.03
QVVHNKLTSCNYNPLYLEADGR-CPDGEVCYSPEKTAEIR	UL128	UL128	intra-protein xl	92	69	23	19.41
VNDKAQYLLGAAGSVPYR-CPDGEVCYSPEKTAEIR	UL128	UL128	intra-protein xl	117	69	48	19.33
QVVHNKLTSCNYNPLYLEADGR-VNDKAQYLLGAAGSVPYR	UL128	UL128	intra-protein xl	92	117	25	17.88

Id	Protein1	Protein2	XLType	AbsPos1	AbsPos2	ID score
YQMCVMKLESWAHVFR-SKAYGGTTEYAASVK	UL130	10F7hc	inter-protein xl	175	54	26.42
SKAYGGTTEYAASVK-IFGAHMVPKQTK	10F7hc	UL130	inter-protein xl	54	154-7	25.21
ADSSPVKAGVETTTPSK-KDQLNR	10F7lc	gH	inter-protein xl	158	283	20.45
ADSSPVKAGVETTTPSK-QAPGKGLEWVGFIR	10F7lc	10F7hc	inter-protein xl	158	43	19.25
CPDGEVCYSPEKTAEIR-SKAYGGTTEYAASVK	UL128	10F7hc	inter-protein xl	69	54	18.9
ADSSPVKAGVETTTPSK-CPDGEVCYSPEKTAEIR	10F7lc	UL128	inter-protein xl	158	69	18.43

Site 3

Id	Protein1	Protein2	XLType	AbsPos1	AbsPos2	deltaAA	ID score
WINLEYDKITR-AKMGYMLQ	UL128	UL128	intra-protein xl	139	165	26	30.14
LNTYALVSKDLASYR-KDQLNR	gH	gH	intra-protein xl	130	283	153	27.83
CPDGEVCYSPEKTAEIR-CYDFKMCNR	UL128	UL128	intra-protein xl	69	47	22	24.84
TTLLLYPTAVDLAKR-VLFKAPYQR	gH	gH	intra-protein xl	404	259	145	24.82
YQMCVMKLESWAHVFR-IFGAHMVPKQTK	UL130	UL130	intra-protein xl	175	154-7	18-21	24.62
TTLLLYPTAVDLAKR-YAVDVLKSGR	gH	gH	intra-protein xl	404	326	78	24.48
LHKTHLASFLSAFAR-TTLLLYPTAVDLAKR	gH	gH	intra-protein xl	452	404	48	22.81
QVVHNKLTSCNYNPLYLEADGR-VNDKAQYLLGAAGSVPYR	UL128	UL128	intra-protein xl	92	117	25	22.43
CPDGEVCYSPEKTAEIR-WINLEYDKITR	UL128	UL128	intra-protein xl	69	139	70	20.53
LVYILSKQNQQHLIPQWALR-TTLLLYPTAVDLAKR	gH	gH	intra-protein xl	428	404	24	19.37
VNDKAQYLLGAAGSVPYR-CPDGEVCYSPEKTAEIR	UL128	UL128	intra-protein xl	117	69	48	18.83
QVVHNKLTSCNYNPLYLEADGR-CYDFKMCNR	UL128	UL128	intra-protein xl	92	47	45	16.55
QTEKHELLVLVK-VLFKAPYQR	gH	gH	intra-protein xl	274	259	15	16.18

Id	Protein1	Protein2	XLType	AbsPos1	AbsPos2	ID score
WINLEYDKITR-KVIWYLSGR	UL128	UL130	inter-protein xl	139	109	29.36

VYACEVTHQGLSSPVTKSFNR-VEPKSCDK	4N10lc	4N10hc	inter-protein xl	206	229	22.81
CPDGEVCYSPEKTAEIR-IFGAHMVPKQTK	UL128	UL130	inter-protein xl	69	154-7	18.54
VYACEVTHQGLSSPVTKSFNRGEC-RVEPKSCDK	4N10lc	4N10hc	inter-protein xl	206	229	16.54

Site 4

Id	Protein1	Protein2	XLType	AbsPos1	AbsPos2	deltaAA	ID score
TASKPSDGNVQISVEDAK-SSTWVKK	UL130	UL130	intra-protein xl	131-45	108-9	22-37	32.22
LNTYALVSKDLASYR-KDQLNR	gH	gH	intra-protein xl	130	283	153	30.2
CPDGEVCYSPEKTAEIR-CYDFKMCNR	UL128	UL128	intra-protein xl	69	47	22	26.96
YQMCVMKLESWAHVFR-IFGAHMVPKQTK	UL130	UL130	intra-protein xl	175	154-7	21	24.29
TASKPSDGNVQISVEDAK-IFGAHMVPKQTK	UL130	UL130	intra-protein xl	131	154-7	23	20.14
QVVHNKLTSCNYNPLYLEADGR-CPDGEVCYSPEKTAEIR	UL128	UL128	intra-protein xl	92	69	23	19.64
VNDKAQYLLGAAGSVPYR-CPDGEVCYSPEKTAEIR	UL128	UL128	intra-protein xl	117	69	48	19.12

Id	Protein1	Protein2	XLType	AbsPos1	AbsPos2	ID score
GLEWVANIKQDGSEK-AKMGYMLQ	10P3 hc	UL128	inter-protein xl	71	165	33.81
GLEWVANIKQDGSEK-WINLEYDKITR	10P3 hc	UL128	inter-protein xl	71	139	30.62
ADSSPVKAGVETTTPSK-AKMGYMLQ	10P3 lc	UL128	inter-protein xl	158	165	27.41
TASKPSDGNVQISVEDAK-RGGTNKR	UL130	UL131A	inter-protein xl	131-45	103	26.42
IFGAHMVPKQTK-KDQLNR	UL130	gH	inter-protein xl	154-7	283	25.4
QDGSEKSYVDSVR-WINLEYDKITR	10P3 hc	UL128	inter-protein xl	77	139	24.41
CPDGEVCYSPEKTAEIR-KVIWYLSGR	UL128	UL130	inter-protein xl	69	109	23.94
QDGSEKSYVDSVR-AKMGYMLQ	10P3 hc	UL128	inter-protein xl	77	165	23.56
TASKPSDGNVQISVEDAK-ADSSPVKAGVETTTPSK	UL130	10P3 lc	inter-protein xl	131-45	158	22.99
ADSSPVKAGVETTTPSK-QAPGKGLEWVANIK	10P3 lc	10P3 hc	inter-protein xl	158	62	22.92
CPDGEVCYSPEKTAEIR-IFGAHMVPKQTK	UL128	UL130	inter-protein xl	69	154-7	21.14
ADSSPVKAGVETTTPSK-IFGAHMVPKQTK	10P3 lc	UL130	inter-protein xl	158	154-7	20.88
YAASSYLSLTPEQWKSHR-VEPKSCDK	10P3 lc	10P3 hc	inter-protein xl	188	246	19.94
ADSSPVKAGVETTTPSK-LNTYALVSKDLASYR	10P3 lc	gH	inter-protein xl	158	130	19.5
VNDKAQYLLGAAGSVPYR-DNSKNSLYLQINSLR	UL128	10P3 hc	inter-protein xl	117	95	18.75
VNDKAQYLLGAAGSVPYR-ADSSPVKAGVETTTPSK	UL128	10P3 lc	inter-protein xl	117	158	18.42
ADSSPVKAGVETTTPSK-CPDGEVCYSPEKTAEIR	10P3 lc	UL128	inter-protein xl	158	69	18.21
LTYSKPHDAATFYCPFLYPSPPR-ADSSPVKAGVETTTPSK	UL130	10P3 lc	inter-protein xl	48	158	16.27

Site 5

Id	Protein1	Protein2	XLType	AbsPos1	AbsPos2	deltaAA	ID score
YQMCVMKLESWAHVFR-IFGAHMVPKQTK	UL130	UL130	intra-protein xl	175	154-7	21	27.89
TASKPSDGNVQISVEDAK-SSTWVKK	UL130	UL130	intra-protein xl	131-45	108-9	23-36	27.43
LNTYALVSKDLASYR-KDQLNR	gH	gH	intra-protein xl	130	283	153	26.56
CPDGEVCYSPEKTAEIR-CYDFKMCNR	UL128	UL128	intra-protein xl	69	47	22	25.04
CPDGEVCYSPEKTAEIR-AKMGYMLQ	UL128	UL128	intra-protein xl	69	165	96	23.81
WINLEYDKITR-AKMGYMLQ	UL128	UL128	intra-protein xl	139	165	26	23.02
QVVHNKLTSCNYNPLYLEADGR-CPDGEVCYSPEKTAEIR	UL128	UL128	intra-protein xl	92	69	23	22.75
YNFVSWYQQHPGKAPK-AGVETTTPSKQSNNK	2C12 lc	2C12 lc	intra-protein xl	44	172	128	22.14
VNDKAQYLLGAAGSVPYR-CPDGEVCYSPEKTAEIR	UL128	UL128	intra-protein xl	117	69	48	21.19
QVVHNKLTSCNYNPLYLEADGR-VNDKAQYLLGAAGSVPYR	UL128	UL128	intra-protein xl	92	117	25	20.16
TASKPSDGNVQISVEDAK-IFGAHMVPKQTK	UL130	UL130	intra-protein xl	131-45	154-7	9-26	19.6
VNDKAQYLLGAAGSVPYR-CYDFKMCNR	UL128	UL128	intra-protein xl	117	47	70	16.98

Id	Protein1	Protein2	XLType	AbsPos1	AbsPos2	ID score
ADSSPVKAGVETTTPSK-LTITKDTSK	2C12 lc	2C12 hc	inter-protein xl	162	73	25.04
TASKPSDGNVQISVEDAK-GGTNKR	UL130	UL131A	inter-protein xl	131-45	103	24.65
ADSSPVKAGVETTTPSK-SSTWVKK	2C12 lc	UL130	inter-protein xl	162	108-9	24.31
CPDGEVCYSPEKTAEIR-LTITKDTSK	UL128	2C12 hc	inter-protein xl	69	73	24.13
LNTYALVSKDLASYR-GGTNKR	gH	UL131A	inter-protein xl	130	103	24.13
ADSSPVKAGVETTTPSK-IFGAHMVPKQTK	2C12 lc	UL130	inter-protein xl	162	154-7	23.03
LTITKDTSK-GGTNKR	2C12 hc	UL131A	inter-protein xl	73	103	22.53
TASKPSDGNVQISVEDAK-LTITKDTSK	UL130	2C12 hc	inter-protein xl	131-45	73	21.19
ADSSPVKAGVETTTPSK-YQMCVMKLESWAHVFR	2C12 lc	UL130	inter-protein xl	162	175	19.31
ADSSPVKAGVETTTPSK-AKMGYMLQ	2C12 lc	UL128	inter-protein xl	162	165	18.79
TASKPSDGNVQISVEDAK-AKMGYMLQ	UL130	UL128	inter-protein xl	131-45	165	17.74
ADSSPVKAGVETTTPSK-CPDGEVCYSPEKTAEIR	2C12 lc	UL128	inter-protein xl	162	69	16.73

Site 6

Id	Protein1	Protein2	XLType	AbsPos1	AbsPos2	deltaAA	ID score
LNTYALVSKDLASYR-KDQLNR	gH	gH	intra-protein xl	130	283	153	29.82
TASKPSDGNVQISVEDAK-SSTWVKK	UL130	UL130	intra-protein xl	131-45	109	23-36	25.14
WINLEYDKITR-AKMGYMLQ	UL128	UL128	intra-protein xl	139	165	26	22.44
QTEKHELLVLVK-VLFKAPYQR	gH	gH	intra-protein xl	274	259	15	21.57
YQMCVMKLESWAHVFR-IFGAHMVPKQTK	UL130	UL130	intra-protein xl	175	154-7	21	20.89
QTEKHELLVLVK-KDQLNR	gH	gH	intra-protein xl	274	283	9	19.42
VNDKAQYLLGAAGSVPYR-CPDGEVCYSPEKTAEIR	UL128	UL128	intra-protein xl	117	69	48	19.2

Id	Protein1	Protein2	XLType	AbsPos1	AbsPos2	ID score
DNSKNTLFLQMNSLR-AKMGYMLQ	7I13 hc	UL128	inter-protein xl	76	165	31.99
VYACEVTHQGLSSPVTKSFNR-VEPKSCDK	7I13 lc	7I13 hc	inter-protein xl	207	225	24.52
VYACEVTHQGLSSPVTKSFNR-GGTNKR	7I13 lc	UL131A	inter-protein xl	207	103	22.6
VYACEVTHQGLSSPVTKSFNRGEC-VEPKSCDK	7I13 lc	7I13 hc	inter-protein xl	207	225	21.65
IFGAHMVPKQTK-EAKVQWK	UL130	7I13 lc	inter-protein xl	154-7	145	20.8
HKVYACEVTHQGLSSPVTK-KDQLNR	7I13 lc	gH	inter-protein xl	190	283	20.07

Site 7

Id	Protein1	Protein2	XLType	AbsPos1	AbsPos2	deltaAA	ID score
QTEKHELLVLVK-KDQLNR	gH	gH	intra-protein xl	274	283	9	31.3
LNTYALVSKDLASYR-KDQLNR	gH	gH	intra-protein xl	130	283	153	28.55
VNDKAQYLLGAAGSVPYR-CPDGEVCYSPEKTAEIR	UL128	UL128	intra-protein xl	117	69	48	22.08
YQMCVMKLESWAHVFR-IFGAHMVPKQTK	UL130	UL130	intra-protein xl	175	154-7	21	21.31
QVVHNKLTSCNYNPLYLEADGR-CPDGEVCYSPEKTAEIR	UL128	UL128	intra-protein xl	92	69	23	20.14

Id	Protein1	Protein2	XLType	AbsPos1	AbsPos2	ID score
CPDGEVCYSPEKTAEIR-KVIWYLSGR	UL128	UL130	inter-protein xl	69	109	27.83
VYACEVTHQGLSSPVTKSFNR-VEPKSCDK	8I21 lc	8I21 hc	inter-protein xl	208	223	17.93
VNDKAQYLLGAAGSVPYR-LNTYALVSKDLASYR	UL128	gH	inter-protein xl	117	130	17.08
